# Mechanisms for Methane and Ammonia Oxidation by Particulate Methane Monooxygenase

**DOI:** 10.1021/acs.jpcb.4c01807

**Published:** 2024-06-08

**Authors:** Per E. M. Siegbahn

**Affiliations:** Department of Organic Chemistry, Arrhenius Laboratory, Stockholm University, SE-106 91 Stockholm, Sweden

## Abstract

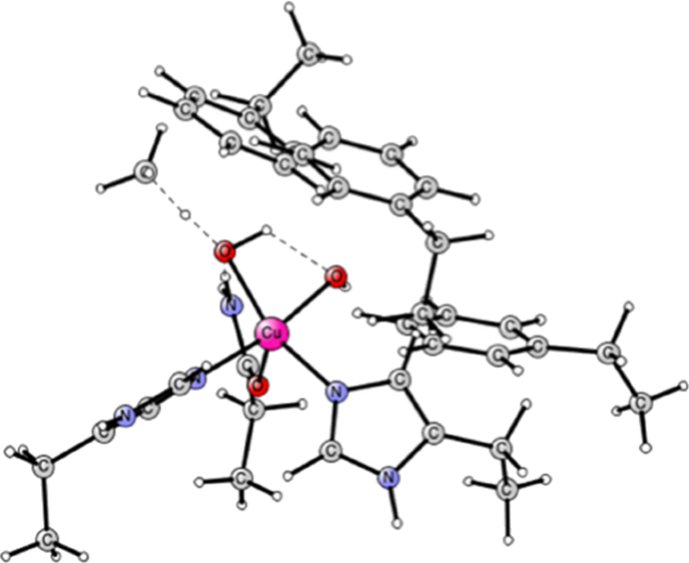

Particulate MMO (pMMO) catalyzes the oxidation of methane to methanol and also ammonia to hydroxylamine. Experimental characterization of the active site has been very difficult partly because the enzyme is membrane-bound. However, recently, there has been major progress mainly through the use of cryogenic electron microscopy (cryoEM). Electron paramagnetic resonance (EPR) and X-ray spectroscopy have also been employed. Surprisingly, the active site has only one copper. There are two histidine ligands and one asparagine ligand, and the active site is surrounded by phenyl alanines but no charged amino acids in the close surrounding. The present study is the first quantum chemical study using a model of that active site (Cu_D_). Low barrier mechanisms have been found, where an important part is that there are two initial proton-coupled electron transfer steps to a bound O_2_ ligand before the substrate enters. Surprisingly, this leads to large radical character for the oxygens even though they are protonated. That result is very important for the ability to accept a proton from the substrates. Methods have been used which have been thoroughly tested for redox enzyme mechanisms.

## 1 Introduction

Methane oxidation in nature is performed by enzymes termed methane mono-oxygenases (MMO’s). There are two classes of these enzymes, one is soluble and is termed sMMO, and the other one is membrane-bound and termed particulate MMO (pMMO); see a recent review.^1^ The X-ray structure of sMMO was determined three decades ago.^2^ It shows an iron dimer bridged by oxygen-derived ligands. The mechanism has been well characterized by experiments and calculations.^3,4^ The mechanism starts by the cleavage of the O_2_ bond, forming a structure with two Fe(IV). The oxygens have a large radical character, and one of them is to abstract hydrogen from methane.

The structure of pMMO has been much more difficult to obtain. After decades of work, an X-ray structure was obtained, but it has unresolved regions.^5,6^ A surprising finding was that there are only copper centers in pMMO. Therefore, the mechanism is unlikely to be the same as in sMMO. It was initially unclear if these centers contained monomers or dimers of copper. A decade after the first structure had been obtained, it became clear that there were only mononuclear copper centers.^7^ A breakthrough came when it became possible to determine a high-resolution cryoEM structure.^8−10^ It has now been concluded that the hydroxylation of methane occurs at the mononuclear Cu_D_ site, which is the active site that is modeled in the present study. The recent review covers all the work, mainly experimental, done on the structure and mechanism of pMMO.^1^

Since the experimentally characterized active site of pMMO is very recent, most theoretical modeling studies, all of them performed earlier, have used the wrong active site. As sMMO starts by cleaving O_2_ using two metal atoms, the initial studies of pMMO started with the possibility that the active site contains a copper dimer.^11−14^ A model of the Cu_B_ site with two copper atoms was used. It has later been shown that this site only contains one copper atom and that the active site is not Cu_B_.^1,6−10,15^ The suggested mechanism is, therefore, not relevant for pMMO. There is only one modeling study that used a mononuclear active site,^16^ but that study modeled the Cu_C_ site, now known not to be the active site. An important difference from the Cu_D_ site is that Cu_C_ has a carboxylate ligand, which strongly affects the oxidation state of copper. An interesting feature of the mechanism suggested is that the electron donor was explicitly included in the model. However, the suggested action of the electron donor is not in agreement with experiments.^1^

The present modeling study of pMMO is the first study that uses the Cu_D_ active site. Methods and models are the same as used in the past years on many redox enzymes, for example, on photosystem II and nitrogenase.^17−20^

## 2 Methods

The methods used here are essentially the same as those used in many previous studies. Density functional theory (DFT) is used with the B3LYP functional.^21^ Instead of the original 20% exact exchange, 15% is used. The choice is based on experience on many redox enzymes.^20^ DFT is used in an unrestricted form. All starting vectors are mixtures of alpha and beta spins, which is important to ensure that the possible closed shell cases are allowed to be mixed. The basis set used for the geometry optimizations, for the Hessian calculations, and for the solvation effects^22^ is lacvp*. For the final point energies, a larger basis set is used with cc-pvtz(-f) for all atoms except copper, where lacv3p* is used. The Mulliken spin populations in the text are taken from the large basis set. For the dispersion effects, the empirical D2 correction was used.^23^ The Jaguar^22^ and Gaussian^24^ programs were used for the calculations.

**Figure 1:**
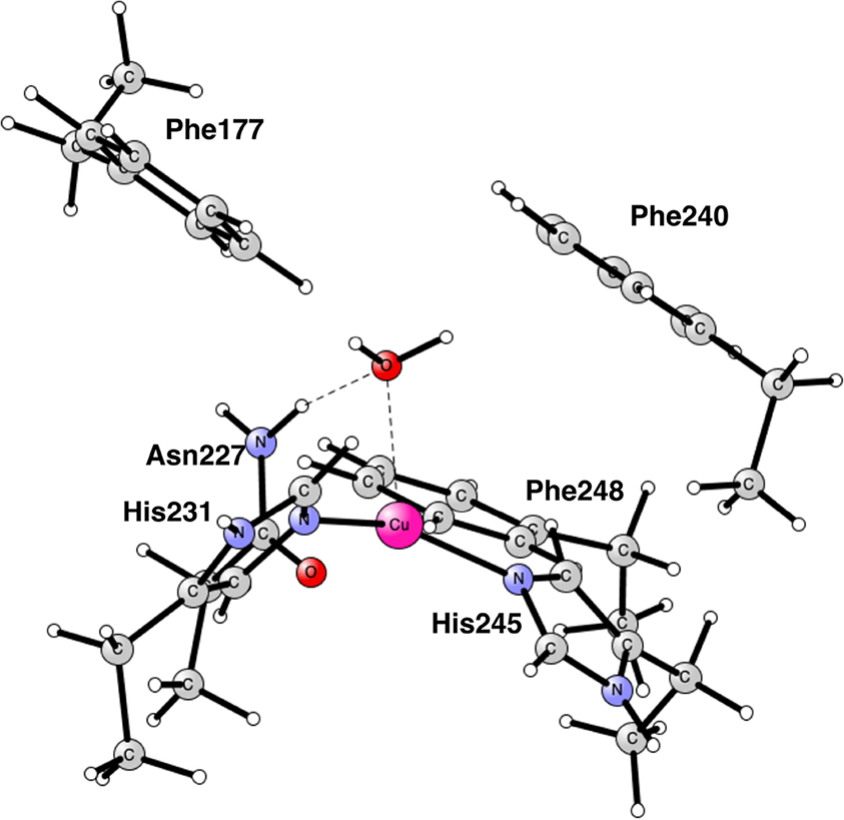
Optimized structure of the Cu_D_ active site in the PmoC subunit of pMMO based on the CryoEM PDB structure 8SR1.^8−10^ The oxidation state of copper is Cu(I).

The cluster model was used to describe the active site.^17^ The starting coordinates were taken from the recent cryoEM structure 8SR1 for the Cu_D_ site,^8−10^ see Figure 1. Copper has two histidines, His231 and His245, and one asparagine, Asn227, ligands. The copper complex is surrounded by three phenyl alanines, Phe177, Phe240, and Phe248. Some backbone atoms are fixed to the cryoEM structure in the optimizations and are marked with a # in the Supporting Information. The backbone atoms fixed are far away from the part of interest of the present study. The experience is that fixing those atoms allows sufficient flexibility for structural changes during the mechanism. The procedure will work unless there are large structural changes that involve the backbone. Such changes are not expected to occur here. The model contains about 100 atoms.

The present procedure and methods have been thoroughly tested for many redox enzymes, showing results which are in general not more than 3 kcal/mol from known experiments.^20^

## 3 Results

This section is divided into two parts. The first section describes the results obtained for the mechanism of methane oxidation. The second concern concerns a similar mechanism for ammonia oxidation. An important energy for the mechanism is the cost of obtaining an electron from the donor and a proton from the medium, which are here considered together as an (H^+^, e^–^) energy. In that way, there are no long-range charge effects from the outside of the model. The physiological donor in nature is not known. Instead, the energy is here estimated from the O–H bond strength, which has been calculated for a model to be 384.1 kcal/mol. The calculated values for the addition of an (H^+^, e^–^) energy can then be obtained for the various intermediates discussed below. Apart from the fact that the electron donor is not known in this case, that procedure has been successfully used for many redox enzymes.^17−20^ It can be added that a duroquinol has been used in another study of the pMMO mechanism, but in a more direct chemical way in a modeling of the Cu_C_ site.^16^ In the present study, the ubiquinol is just used as an electron donor, which can be far away from the active site. The proton is taken from the nearby surroundings of the cofactor.

The first decision of the mechanism is to assign an oxidation state for copper. In the CryoEM structure of the active Cu_D_ site, all the amino acids around copper are neutral. That strongly indicates a copper oxidation state of Cu(I). In contrast, the Cu_C_ site, which was previously believed to be the active site, copper has one negative ligand, leading to a probable Cu(II) oxidation state in that case, which is a quite significant difference. A Cu(I) active site for Cu_D_ is in agreement with EPR measurements.^1,10^

**Figure 2:**
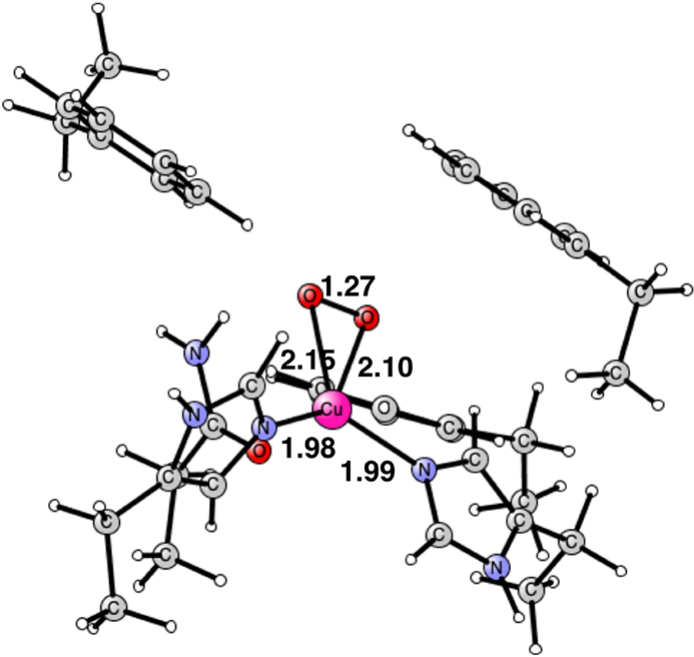
Optimized Cu_D_ structure with bound O_2_. The oxidation state of copper is Cu(II). The state is a triplet. Distances are given in Å.

The mechanism for methane oxidation starts out from the Cu_D_ structure in Figure 1. It was found that a water molecule, not seen in the experimental structure, is bound close to copper; see the figure. The binding energy is 4.9 kcal/mol with a distance of 2.49 Å to copper. The first step in the mechanism is to bind to O_2_ after removing the water. The resulting structure is shown in Figure 2, which is a triplet Cu(II) state. Copper has a spin of 0.33, slightly lower than that for a normal Cu(II) state. There is a large spin on O_2_ with one spin being 0.77 and the other one 0.88. The charge on O_2_ is −0.2. The O–O bond distance is 1.27 Å, and the Cu–O distances are 2.10 and 2.15 Å. The corresponding Cu(II) singlet state is only +1.3 kcal/mol higher in energy. The spin on copper is in that case +0.43 and those on the two oxygens are −0.26 and −0.27, respectively. The closed shell Cu(III) state is much higher in energy. With the loss of translational entropy of 9.3 kcal/mol, the addition of O_2_ is endergonic by +2.5 kcal/mol. Since the removal of water is endergonic by +4.9 kcal/mol, the step is altogether endergonic by +7.4 kcal/mol.

The next step in the mechanism has in most studies been assumed to be the cleavage of O_2_ to form an oxygen with large radical character. That is not the case here. The O–O cleavage has a very high barrier and a large endergonicity. Instead, (H^+^, e^–^) is added to O_2_, which is exergonic by −0.9 kcal/mol, using the cost for cleaving the O–H bond in the ubiquinol donor. Copper stays Cu(II) with a spin of +0.50. The spins on the oxygens are still significant, +0.27 and +0.12. The spin state is a doublet. The O–O bond distance is 1.44 Å.

The energy required to obtain a second (H^+^, e^–^) is here taken to be the same as for the first one, with 384.1 kcal/mol. However, there is also the possibility that the energy requirement is much smaller if (H^+^, e^–^) is taken from the same ubiquinol as for the first one, forming a ubiquinone. Only a detailed study of the electron donor mechanism can determine which of these two possibilities is used in vivo.

**Figure 3:**
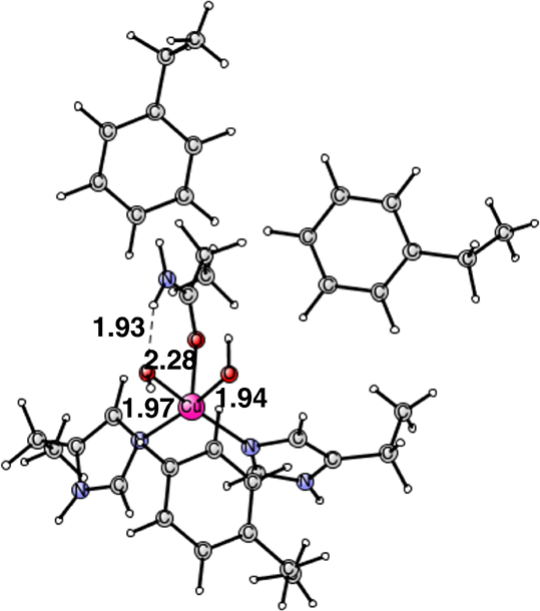
Optimized Cu(II) structure after the addition of two (H^+^, e^–^). The spin on copper is +0.64 and the spins on the two oxygens are −0.43 and −0.40. The distance between the oxygens is 2.19 Å. It is a singlet state. The closed shell Cu(III) structure is similar but with an O–O distance of 2.51 Å. It has no spin. Distances are given in Å.

The addition of the second (H^+^, e^–^) has several interesting consequences, see Figure 3. The most important one is that there is a large increase in the O–O bond distance to 2.19 Å. The O–O distance in the bound O_2_ in Figure 2 is only 1.27 Å and the one for the previous state, with one (H^+^, e^–^) added, is 1.44 Å. The spin on copper is 0.64. The spins on the oxygens are surprisingly large, with −0.43 and −0.40, even though they are protonated. This is required for the next step, when one of the oxygens should abstract a proton from methane. The structure can be assigned as Cu(II) with a bound O_2_- radical. It is a singlet state. The exergonicity to form this Cu(II) state is −12.4 kcal/mol. The spin state obtained for the Cu(II) solution suggests that the Cu(III) state might be low-lying. Indeed, the closed shell Cu(III) state without spin on copper and the oxygens is −6.0 kcal/mol lower than that of the Cu(II) state. The O–O bond is now fully cleaved at a distance of 2.51 Å. The exergonicity for the addition of the second (H^+^, e^–^) forming the Cu(III) state is quite large with −18.4 kcal/mol. It can be added that for the mechanism of methane oxidation, a precise value of the energy to obtain a (H^+^, e^–^) does not matter as long as the addition is exergonic.

**Figure 4:**
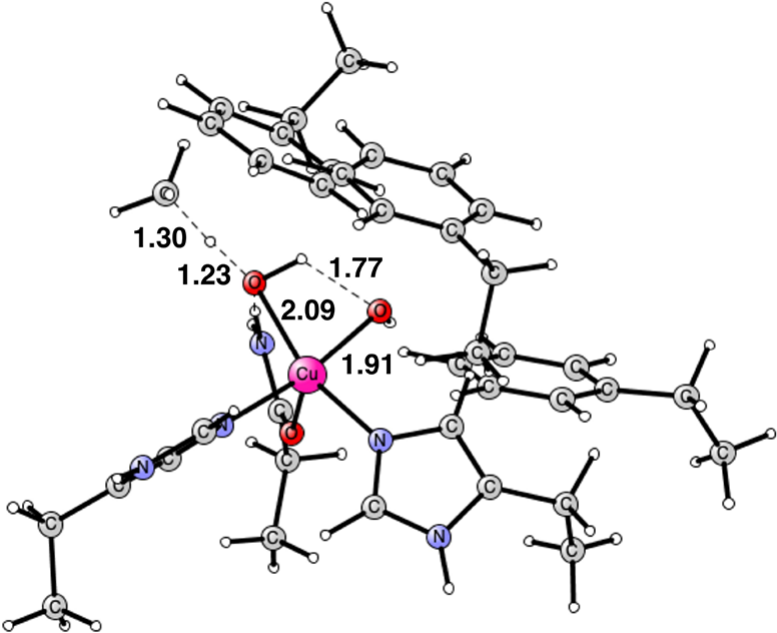
Optimized TS structure for the abstraction of hydrogen from methane. The spin on Cu is 0.58, on methyl is −0.54, and on OH is −0.34. The barrier is 17.9 kcal/mol. It is a singlet state. Distances are given in Å.

At this stage, methane is added. The state that can activate methane is the Cu(II) state, where the oxygens have very high spins. It is a singlet state. A TS for the hydrogen abstraction from methane is shown in Figure 4. The distance from the hydrogen being abstracted to CH_3_ is 1.30 Å and to oxygen 1.23 Å. The spin on that oxygen changed from −0.43 to −0.34. The spin on CH_3_ is −0.54. It may be surprising that the spin on the other oxygen on copper changes much more from −0.40 to +0.10. The spin on copper hardly changes from 0.64 to 0.58, still indicating a Cu(II) state. There is a loss of translational entropy when a free methane forms the TS. If all that entropy is lost, it means a cost of 8.7 kcal/mol, obtained from a particle in a box. However, it seems from the structure that some entropy is kept, here estimated to be 3.0 kcal/mol. The barrier then increased to 17.9 kcal/mol. There is a favorable dispersion effect of −4.0 kcal/mol, lowering the barrier coming from the interactions with the phenyl alanines, which contribute −6.1 kcal/mol.

**Figure 5:**
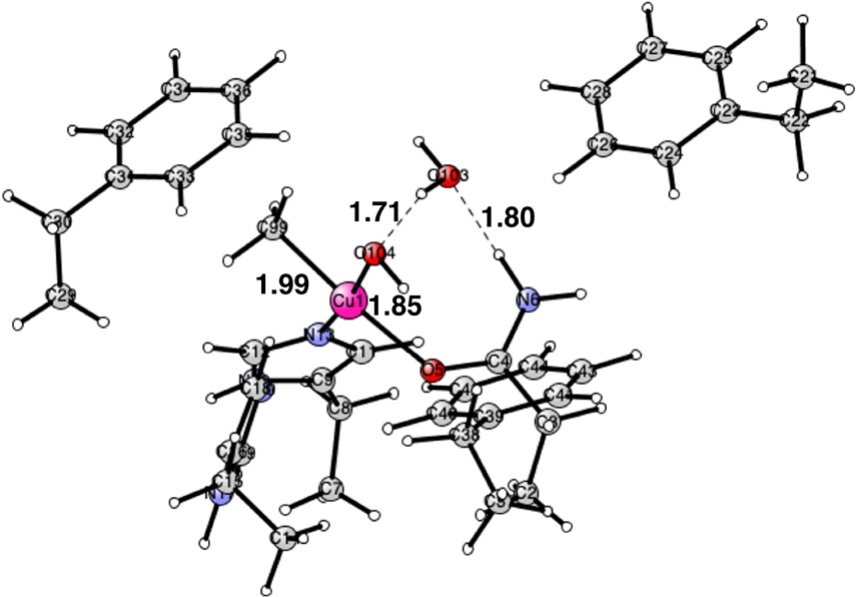
Optimized structure for the binding of methyl to copper. The oxidation state of copper is Cu(III). It is a singlet state. Distances are given in Å.

After the hydrogen abstraction, water is formed bound to copper. The energy decreases from the TS by −13.5 kcal/mol. The methyl is now a fully developed radical with a spin of −1.09. Copper is still Cu(II) with a spin of 0.60. The methyl was then followed in short steps until its endpoint. It turned out that water is pushed away and instead methyl binds to copper, see Figure 5, with a large gain of energy of −15.3 kcal/mol, and a short Cu–C distance of 1.99 Å. At this point, the cofactor has no spin, indicating a Cu(III) state without spin on methyl. A surprising result is that the charge population on the bound methyl is +0.4. However, charges obtained from a population analysis should be regarded with skepticism since there are overlapping orbitals.

**Figure 6:**
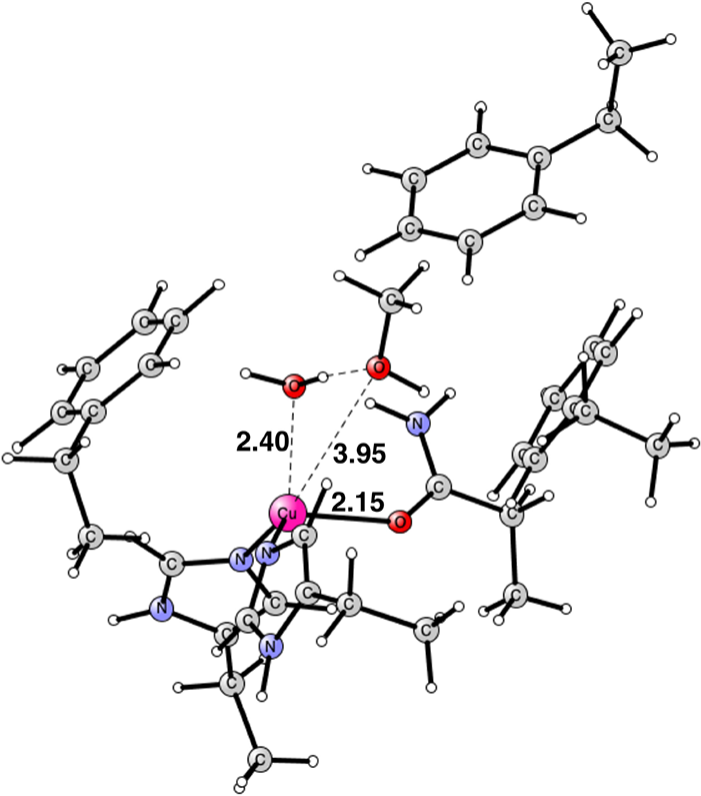
Optimized structure of the methanol product. The oxidation state of copper is Cu(I). It is a singlet state. Distances are given in Å.

**Figure 7:**
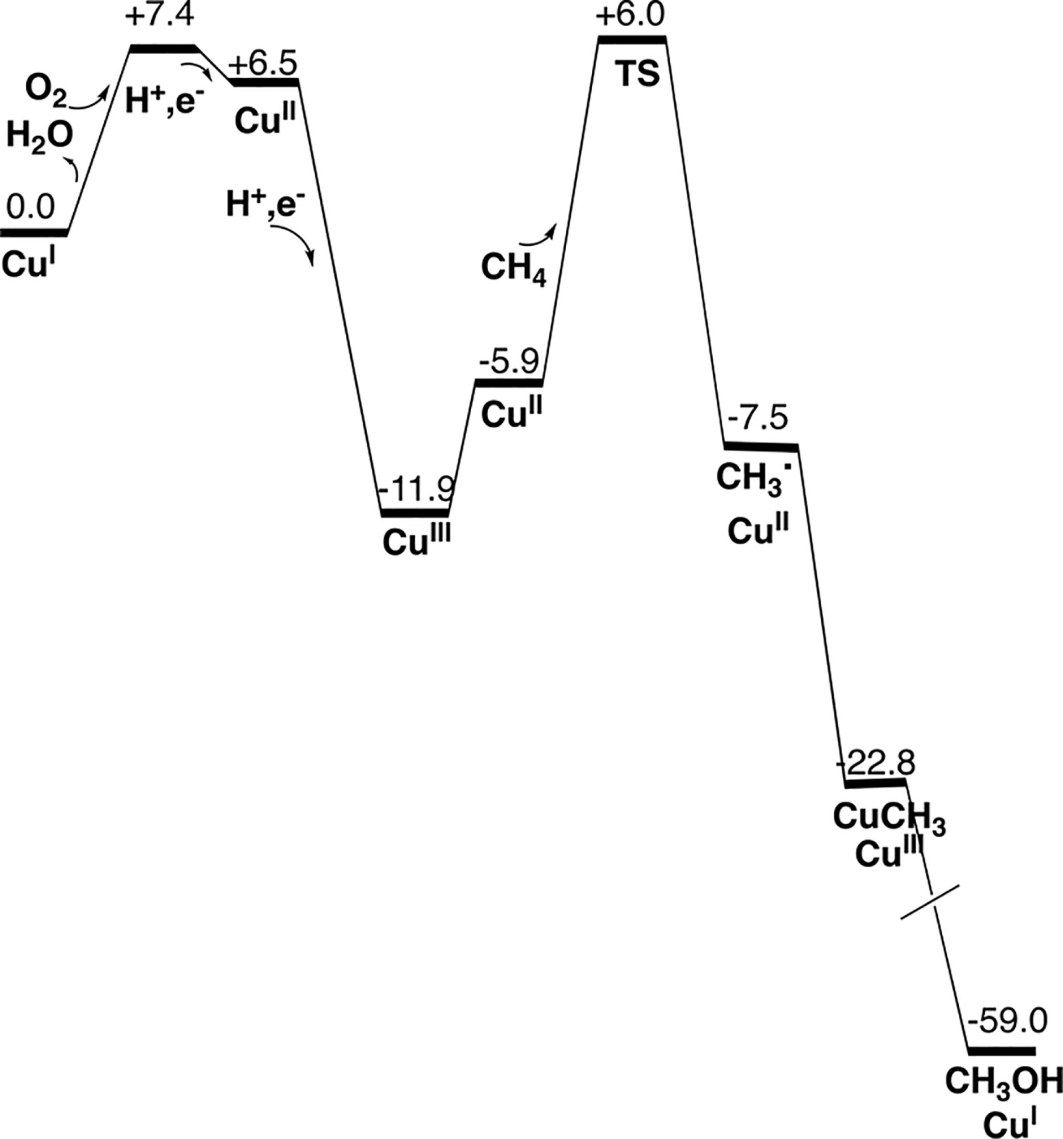
Energy diagram for the mechanism of methane oxidation in pMMO. The free energies are given in kcal/mol.

The final step is the formation of the C–O bond of methanol. At most, a very small barrier was found in this step even though there is electron transfer involved. Copper goes from Cu(III) to Cu(I), which occurs very smoothly via overlapping orbitals. The step is very exergonic, −36.2 kcal/mol. The optimal structure of the product is shown in Figure 6. Methanol has lost its coordination with a distance to copper of 3.95 Å. Water forms a weak bond with a distance of 2.40 Å, just as the one in the starting point in Figure 1. The binding of methanol is weak and similar to that of a water molecule at the same position. The energy diagram for the oxidation of methane is shown in Figure 7. The rate-limiting step is the abstraction of hydrogen from methane with a barrier of 17.9 kcal/mol, which is a feasible size. A striking feature, but which is not unexpected, is the very large exergonicity. A surprising result is the strong bonding of methyl to copper, forming a Cu(III) state.

**Figure 8:**
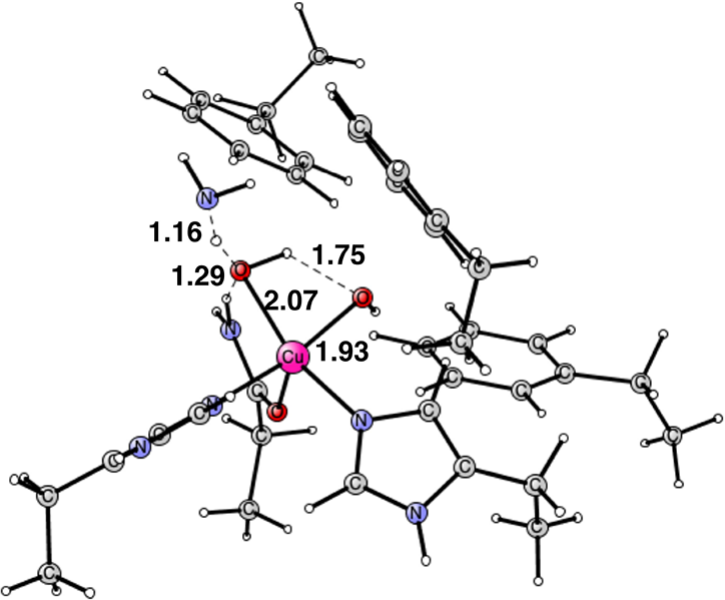
Optimized TS structure for hydrogen abstraction from ammonia. The spin on Cu is 0.57, on NH_2_ is −0.72, and on OH is −0.21. The barrier is 14.4 kcal/mol. It is a singlet state. Distances are given in Å.

The mechanism for ammonia oxidation is similar to the one for methane, but there are still rather large differences in the relative energies. The first part of the mechanism before ammonia becomes activated is identical. The TS for hydrogen abstraction from ammonia is shown in Figure 8. The TS is earlier than that for methane, with an N–H bond of 1.16 Å compared to the C–H bond of 1.30 Å. The barrier height counted from the resting Cu(III) state is 14.4 kcal/mol for ammonia compared to 17.9 kcal/mol for methane. The Cu atom is Cu(II) in both cases, with almost the same spin population. The amino group has a spin of −0.72 compared to that of the methyl group of −0.54.

The hydrogen abstraction results in a free NH_2_ radical. That radical is less stable than the methyl one with an energy of +9.4 kcal/mol with respect to the resting state compared to an energy of +4.4 kcal/mol for methyl. A water bound to copper is formed after the hydrogen abstraction. The Cu–O distance is 2.18 Å, which is shorter than the one in the methane case of 2.30 Å. This difference plays some role in the next step, where the water molecule needs to be removed.

**Figure 9:**
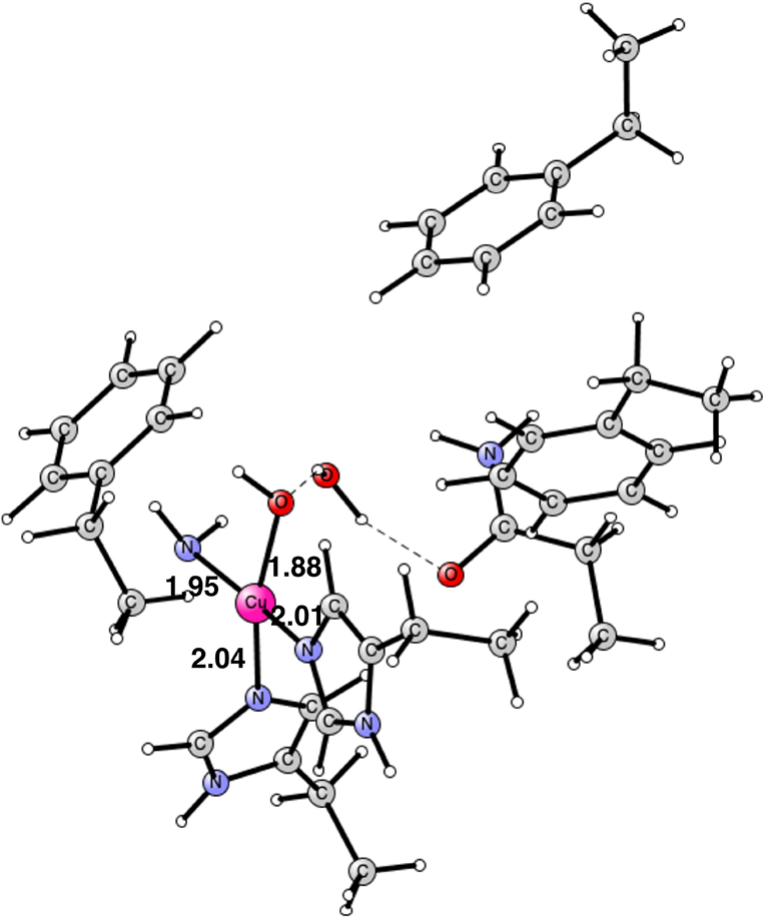
CuNH_2_ intermediate product. The oxidation state of copper is Cu(II). It is a singlet state. Distances are given in Å.

In the next step, the difference between the two mechanisms becomes the largest. The methyl radical follows an unproblematic pathway to copper and forms a Cu(III) state for the cofactor. A straightforward pathway for the amino radical, on the other hand, does not lead to a Cu(III) state but rather to a quite unstable Cu(II) complex. After rather many attempts to improve the energy for the amino bound state, a reconstruction around copper was found, see Figure 9. The reason for the reconstruction is that there is a strong preference for a square planar coordination of Cu(II). Cu(III) does not have such a distinct preference. Once a square planar coordination was achieved with the amino group strongly bound to copper with a distance of 1.95 Å, the complex becomes quite stable with an energy of −1.7 kcal/mol with respect to the resting state, However, the corresponding structure for the methyl case is much more stable with −10.9 kcal/mol due to the stability of Cu(III) in that case.

**Figure 10:**
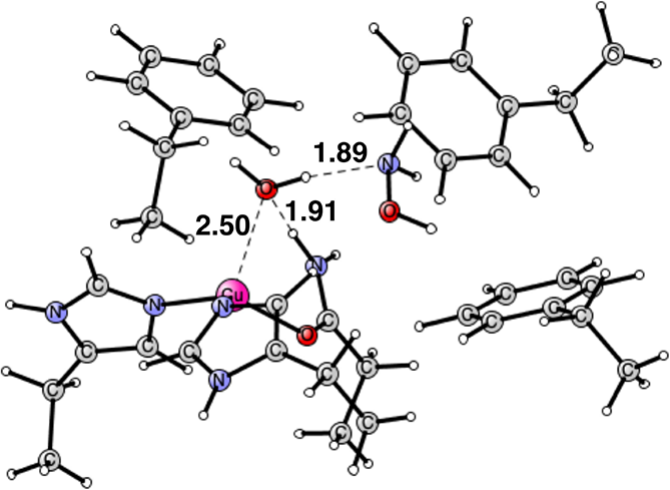
Optimized structure of the hydroxylamine product. The oxidation state of copper is Cu(I). It is a singlet state. Distances are given in Å.

In the final step of the mechanism, the NH_2_OH product is formed, see Figure 10. For a smooth formation of the N–O bond, it is required that both the amino and hydroxide groups have strong bonds to copper. The reason is that the bond formation requires an electron transfer to copper, which is strongly dependent on these distances. Many attempts were made to directly move the free NH_2_ radical toward the hydroxide and form the NO bond, without forming the strong Cu–NH_2_ bond, but that turned out not to be possible.

**Figure 11:**
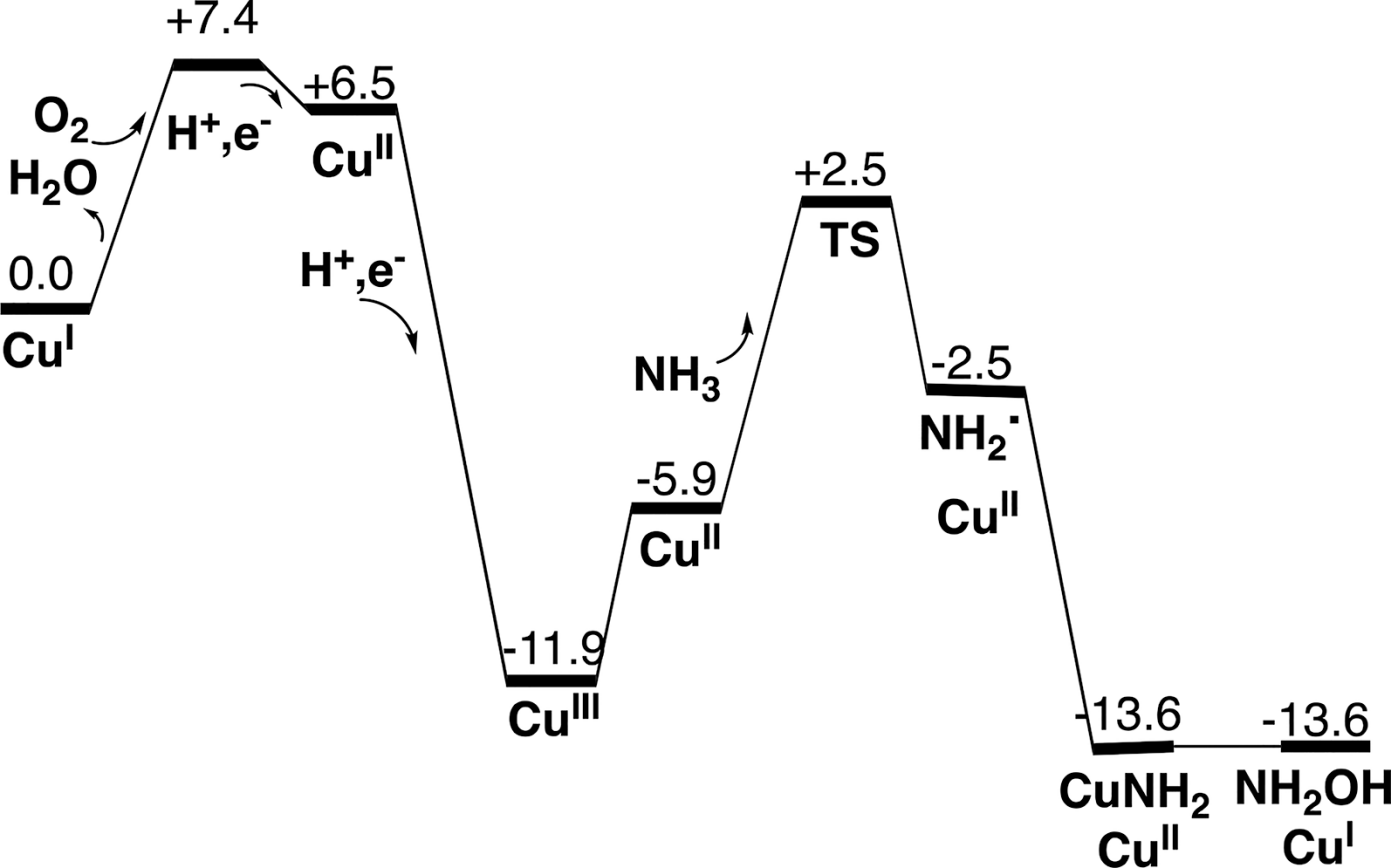
Free-energy diagram for the mechanism of ammonia oxidation in pMMO. The free energies are given in kcal/mol.

The final product for the ammonia oxidation is very similar to the one for the case of methane; see Figure 10. The hydroxylamine automatically moves away from copper. The final distance from the oxygen of hydroxylamine to copper is 4.10 Å, compared to 3.95 Å for the corresponding distance for methanol. There is a hydrogen bond to the water in both cases, with the water weakly bound to copper. The full energy diagram for ammonia oxidation by pMMO is shown in Figure 11. The most striking difference to the one for methane occurs at the end of the mechanism, where the formation of methanol is very exergonic and that for hydroxylamine is only weakly exergonic.

## 4 Conclusions

The mechanisms for the oxidation of methane and ammonia by pMMO have been described. For a long time, it was assumed that the activation of methane should require at least two metal atoms. Therefore, when the experimental cryoEM structure was determined and clearly showed that the active site has only one copper atom, it came as a big surprise.^1,5−10^ The present study is the first in which the Cu_D_ active site, suggested experimentally, has been used in a quantum chemical modeling study. The main step forward to an understanding of the mechanism was taken when it was realized that there are two proton coupled electron transfer steps before methane enters. That resulted in a cleavage of the O–O bond and formation of a Cu(II) state. Surprisingly, and very importantly, the spins on the hydroxides formed are very large, which is required for the next step when methane enters. A Cu(III) state is lower in energy than the Cu(II) state, However, the formation of the Cu(III) state is unimportant at that stage.

The formation of the Cu(II) and Cu(III) states is common to methane and ammonia oxidation. The next step in both cases is hydrogen abstraction from the substrate, which is made possible by the large radical character of the hydroxides. For methane, that resulted in the formation of another Cu(III) state. In the ammonia case, the corresponding intermediate is a Cu(II) complex. Therefore, for the ammonia case, it is important that the copper complex become square planar. In both cases, a complex with strong Cu–C (or Cu–N) and Cu–OH bonds is required to allow efficient electron transfer to copper when the C–O (or C–N) bond is formed.

The methods used here have been thoroughly tested for redox mechanisms for enzymes, with highly accurate results in all cases so far. This is also a requirement for solving the mechanism for pMMO.^17−20^

## Supporting Information Available

Coordinates for all structures discussed in the text (PDF)
