# Supplementary material for: Mechanisms for Methane and Ammonia Oxidation by Particulate Methane Monooxygenase
Source: J Phys Chem B. 2024 Jun 8;128(24):5840–5. doi: 10.1021/acs.jpcb.4c01807 (PMC11194816; doi:10.1021/acs.jpcb.4c01807)
Supplement: Supplementary file 1 — jp4c01807_si_001.pdf [file jp4c01807_si_001.pdf]

Supporting Information:

The Mechanisms for Methane and Ammonia Oxidations by Particulate Methane Monooxygenase (pMMO)

Per E. M. Siegbahn*

Department of Organic Chemistry, Arrhenius Laboratory, Stockholm University,

SE-106 91, Stockholm, Sweden. Email:per.siegbahn@su.se

The B3LYP optimized structure in **Figure 1.** # means fixed atom
Energies: E= -2062.652886, solv = -0.055538, disp = -77.86 Z_0 = 544.38

Cu1	118.3464066620	124.8008441622	131.9894781880
C2	116.5619986405#	129.0119914532#	134.2799996190#
C3	115.8178991771	127.7641410756	134.7634207637
C4	116.2425420205	126.4933596351	134.0207073884
O5	117.3242050156	126.4132144672	133.4098705552
N6	115.3970265887	125.4419102609	134.0936605420
C7	117.9430038738#	129.3260022909#	128.0959970129#
C8	116.9149220638	128.1582377204	127.9522020107
C9	117.1603615434	126.9192425683	128.7422341417
N10	117.3610210194	125.6943160886	128.1266146718
C11	117.2636911639	126.6582211877	130.0828055648
C12	117.5831829341	124.7582078627	129.0794770253
N13	117.5297204104	125.3144805667	130.2794002716
C14	122.0159978587#	125.9889974343#	136.4929940504#
C15	122.3268384828	124.6561339980	135.7578087490
C16	121.6462376507	124.5745532436	134.4346835082
N17	122.2009610158	124.9957546036	133.2389105285
C18	120.3471783291	124.2635304844	134.1281040301
C19	121.2476205720	124.9360894928	132.2769128636
N20	120.1073476244	124.4997184967	132.7905186395
C21	109.3710048762#	123.1240018194#	138.9649942582#
C22	109.5941156195	124.2391927599	137.9088048510
C23	110.6193105912	123.8962426319	136.8447007146
C24	111.9887499207	123.9015586303	137.1569909981
C25	110.2454678151	123.5728555806	135.5326722619
C26	112.9529566043	123.5922027040	136.1958119372
C27	111.2037574429	123.2659331653	134.5629030614
C28	112.5618667960	123.2735982834	134.8902158978
C29	122.3130003907#	121.1350043644#	132.0729965331#
C30	121.7250632360	119.7397724742	132.3705783105
C31	120.2238361382	119.8232137506	132.5717115803
C32	119.3422777462	119.7142879950	131.4844343648
C33	119.6810922499	120.0651709459	133.8428459520
C34	117.9620717774	119.8265172454	131.6614603017
C35	118.3012754562	120.1804911957	134.0299925246
C36	117.4329877806	120.0552627238	132.9386625007
C37	119.6179982059#	125.6200042557#	141.7319941595#
C38	119.2530556631	124.5539464918	140.6596203181
C39	118.2046454914	124.9647623530	139.6464061358
C40	118.4720686269	124.9807076764	138.2719797445
C41	116.9027866661	125.2791564188	140.0712336035
C42	117.4693958791	125.2761393329	137.3436389967
C43	115.8974283865	125.5752839193	139.1530364735
C44	116.1754136026	125.5641423170	137.7831470596
H45	117.6441958318#	129.9707658536#	128.7995432298#
H46	117.8513697184#	129.7498307758#	127.1948926568#

H47	118.9651296243	128.9801807466	128.2636672493
H48	115.9146663196	128.5490646757	128.1787670833
H49	116.8754138185	127.8668526594	126.8948187808
H50	117.3310754239	125.5291420096	127.1295508045
H51	117.1774890675	127.3297428740	130.9233939129
H52	117.7894690735	123.7202297253	128.8652399857
H53	116.3262775892#	129.8431699261#	134.7835684610#
H54	116.3895100765#	129.1526954553#	133.3050842288#
H55	117.6392091589	128.8483522322	134.3937361544
H56	116.0160981348	127.5919846283	135.8289072739
H57	114.7308759848	127.8868641760	134.6707119129
H58	122.0185852296#	125.7875134452#	137.4724878853#
H59	121.1177291401#	126.3387999866#	136.2270058241#
H60	122.7671666411	126.7550697676	136.2740824988
H61	123.4081000971	124.5070859138	135.6558261648
H62	121.9648704466	123.8199448097	136.3667766078
H63	123.1573416163	125.2945091756	133.1045313461
H64	119.5682544386	123.9079679501	134.7856098834
H65	121.4181676853	125.2029450050	131.2452406809
H66	122.0339375581#	121.7673967159#	132.7956243848#
H67	121.8908622777	121.5129425085	131.1319363121
H68	123.3089601933#	121.1488699750#	131.9842088513#
H69	121.9562883532	119.0490228141	131.5515324224
H70	122.1937405460	119.3269284073	133.2725099182
H71	116.3561610355	120.0743120770	133.0924298621
H72	117.2967065913	119.6987860258	130.8111847214
H73	119.7420649001	119.5142875209	130.4927806929
H74	120.3454317250	120.1418209893	134.7005669504
H75	117.9017901771	120.3322957174	135.0294147448
H76	119.7774565414#	125.1411506054#	142.5952910503#
H77	118.8836745233#	126.2880934066#	141.8520973853#
H78	120.5229714767	126.1689431855	141.4495193026
H79	120.1589240478	124.2253541312	140.1368866934
H80	118.8741569363	123.6629565819	141.1789746998
H81	116.6711915903	125.2708568694	141.1339654163
H82	114.8947690655	125.8046065181	139.5038679817
H83	115.3856878671	125.7749337985	137.0691321348
H84	119.4760482177	124.7440723079	137.9257578560
H85	117.7000309269	125.2816318029	136.2810557051
H86	110.2531353487#	122.7250810654#	139.2154088068#
H87	108.7333159108	122.3269734373	138.5674055637
H88	108.9442664050#	123.5248261710#	139.7756956018#
H89	108.6414082757	124.5017958914	137.4347148817
H90	109.9327961923	125.1431869025	138.4330817854
H91	112.3038201393	124.1513573501	138.1682125519
H92	114.0045644907	123.5839925514	136.4738224785
H93	109.1911224968	123.5651231286	135.2660009009
H94	110.8869352383	123.0169727508	133.5535403778
H95	113.3067132464	123.0140090260	134.1409508418
H96	115.6856179512	124.5588251329	133.6762721832
H97	114.5103201312	125.4849078568	134.5778137193
O98	116.6238788438	123.1403129981	132.6901647480
H99	117.0486928182	122.2902016453	132.9175721892
H100	116.2551375632	123.0072489206	131.8028446217

The B3LYP optimized structure in **Figure 2.**
Energies: E= --2136.525346, solv = -0.055627, disp = -76.07 Z_0 = 532.29

Cu1	118.2123432653	124.7225164720	132.1970846498
C2	116.5619972780#	129.0119925822#	134.2799994503#
C3	115.7609820927	127.8051753882	134.7791865307
C4	116.0557156251	126.5336858443	133.9939242649
O5	117.1623920572	126.3332788327	133.4592238923
N6	115.0566831343	125.6267241963	133.9278351942
C7	117.9430023680#	129.3260032154#	128.0959949196#
C8	116.9829390023	128.1142979525	127.9582979443
C9	117.2846289594	126.9264128477	128.8036815062

N10	117.1664080541	125.6344732912	128.3164272040
C11	117.6990846543	126.7762433466	130.0996530704
C12	117.4996863793	124.7641116598	129.2955103522
N13	117.8284418140	125.4322093020	130.3886157358
C14	122.0159983514#	125.9889974120#	136.4929949974#
C15	122.3783239864	124.7105991622	135.6944555249
C16	121.7034347270	124.7280117031	134.3667021497
N17	122.1104919812	125.4914537283	133.2838134494
C18	120.4681700534	124.2565092872	134.0175457484
C19	121.1331176652	125.4701567142	132.3461044961
N20	120.1192232141	124.7332886845	132.7701229883
C21	109.3710034172#	123.1240012352#	138.9649951702#
C22	109.5821361341	124.2478338737	137.9165730971
C23	110.5507307213	123.9008492603	136.8030082534
C24	111.9342820699	123.8978341783	137.0443933678
C25	110.1072640002	123.5820109098	135.5117965462
C26	112.8452088918	123.5829507843	136.0339008995
C27	111.0116281117	123.2693102663	134.4936436744
C28	112.3848607607	123.2675378523	134.7503919071
C29	122.3130031737#	121.1350028056#	132.0729975364#
C30	121.7652802384	119.7231051443	132.3698436767
C31	120.2565136004	119.7303548496	132.5093679045
C32	119.4299771219	119.5791840774	131.3861073120
C33	119.6480160325	119.9380229534	133.7561679138
C34	118.0387488414	119.6331949607	131.5032404096
C35	118.2575812987	119.9940853810	133.8795862471
C36	117.4469848548	119.8420048802	132.7515539888
C37	119.6179969578#	125.6200035674#	141.7319946433#
C38	119.2608671024	124.5438785867	140.6718808473
C39	118.3071512185	124.9530962536	139.5677263722
C40	118.6212185486	124.7476507407	138.2175296478
C41	117.0306588092	125.4480325331	139.8824416444
C42	117.6791779283	124.9834327528	137.2123528960
C43	116.0849874964	125.6847738875	138.8864289956
C44	116.4014085769	125.4360160806	137.5478126760
H45	117.6441961009#	129.9707649099#	128.7995442092#
H46	117.8513707682#	129.7498313420#	127.1948928161#
H47	118.9790632317	129.0171014450	128.2580666723
H48	115.9539498406	128.4532887062	128.1418971097
H49	116.9965262112	127.7844297268	126.9114322134
H50	116.8823976082	125.3856448702	127.3781163504
H51	117.8924623055	127.5299059831	130.8474374263
H52	117.4911438637	123.6903836225	129.1831440036
H53	116.3262783757#	129.8431700352#	134.7835686496#
H54	116.3895104733#	129.1526951280#	133.3050841114#
H55	117.6324264094	128.8212573499	134.4077775705
H56	116.0073677370	127.5961529451	135.8279763232
H57	114.6833202223	128.0086446659	134.7474597929
H58	122.0185851524#	125.7875138580#	137.4724879705#
H59	121.1177291534#	126.3387996304#	136.2270053113#
H60	122.7499370019	126.7821758971	136.3055512626
H61	123.4646684815	124.5977652854	135.6003489870
H62	122.0235475596	123.8233349615	136.2297540102
H63	122.9923702412	125.9796247588	133.2026732330
H64	119.8056437249	123.6233560634	134.5868390836
H65	121.1928660647	125.9825912137	131.3978629644
H66	122.0339357838#	121.7673956406#	132.7956240767#
H67	121.8866746889	121.5022837032	131.1296729117
H68	123.3089600793#	121.1488713911#	131.9842077920#
H69	122.0633520271	119.0349865920	131.5696821318
H70	122.2200615447	119.3432412425	133.2935734686
H71	116.3651319684	119.8689152664	132.8474202195
H72	117.4168416743	119.4913182555	130.6228849005
H73	119.8816389821	119.3998797148	130.4123993389
H74	120.2709585029	120.0373997305	134.6430792547
H75	117.8070727371	120.1386226321	134.8579046166
H76	119.7774573835#	125.1411508182#	142.5952910128#
H77	118.8836747916#	126.2880937394#	141.8520971756#
H78	120.5225628404	126.1686308078	141.4469692653
H79	120.1802302048	124.1422735296	140.2296375592
H80	118.7958855777	123.6962165903	141.1952290298

H81	116.7625239751	125.6147756822	140.9230384681
H82	115.0951015891	126.0436373842	139.1556637063
H83	115.6496786788	125.5710595886	136.7769800836
H84	119.6056666073	124.3648164353	137.9561037331
H85	117.9355365226	124.8017553199	136.1715775752
H86	110.2531356594#	122.7250814944#	139.2154083945#
H87	108.7326126218	122.3280362765	138.5663027184
H88	108.9442667326#	123.5248261947#	139.7756957629#
H89	108.6160565613	124.5391390813	137.4881992523
H90	109.9628404342	125.1352098001	138.4406019494
H91	112.3023617219	124.1434538567	138.0385837251
H92	113.9095493511	123.5651890944	136.2575390047
H93	109.0404542856	123.5813827737	135.3003633500
H94	110.6419399462	123.0214369927	133.5022473256
H95	113.0856418878	122.9951814181	133.9640375576
H96	115.2271122311	124.7311576002	133.4876447564
H97	114.1694529396	125.7539836108	134.3960907193
O98	116.6115378588	123.3303878205	132.5194670821
O99	117.4767463419	123.2023938822	133.4444891775

The B3LYP optimized structure for CuOOH.
Energies: E= --2137.143450, solv = -0.059216, disp = -78.97 Z_0 = 540.38

Cu1	117.7886438026	124.5962751233	132.4314939531
C2	116.5619979659#	129.0119920024#	134.2799994710#
C3	115.6959523727	127.8545290634	134.8101521021
C4	115.9379999285	126.5578305020	134.0634644320
O5	117.1019482204	126.2014691915	133.7738245262
N6	114.8710142925	125.8007137960	133.7468069445
C7	117.9430029952#	129.3260029607#	128.0959957012#
C8	117.0330522623	128.0748781430	127.9867119756
C9	117.2432344747	126.9210912474	128.9233255156
N10	116.8722378338	125.6413944562	128.5289065993
C11	117.6945598446	126.7638225130	130.2102666595
C12	117.0934713022	124.7798202093	129.5450366709
N13	117.5965934668	125.4299139963	130.5813997495
C14	122.0159981556#	125.9889976062#	136.4929946840#
C15	122.2825790310	124.6919860413	135.6701565315
C16	121.4468973434	124.6667697238	134.4274318024
N17	121.8422675805	125.1706265128	133.1979576327
C18	120.1229125844	124.3516802287	134.2596667186
C19	120.7843733990	125.1481110207	132.3524207865
N20	119.7215945257	124.6620635092	132.9736154691
C21	109.3710040429#	123.1240013121#	138.9649947835#
C22	109.5756020922	124.2445180443	137.9119587582
C23	110.5409957113	123.8974241476	136.7956036685
C24	111.9267119752	123.9410945957	137.0207013629
C25	110.0937487059	123.5337280041	135.5177712886
C26	112.8344400323	123.6322550748	136.0063228356
C27	110.9966090588	123.2241960381	134.4968758311
C28	112.3721762621	123.2730208125	134.7356432425
C29	122.3130018044#	121.1350035876#	132.0729970655#
C30	121.7784343090	119.7074771986	132.3163662858
C31	120.3025237372	119.5591415346	131.9972789739
C32	119.8712645903	118.6376764346	131.0337662607
C33	119.3335512392	120.3519240566	132.6335620333
C34	118.5177411486	118.5078019902	130.7114970363
C35	117.9792701001	120.2295383519	132.3145409853
C36	117.5671399020	119.3056328786	131.3492411990
C37	119.6179976743#	125.6200040494#	141.7319945415#
C38	119.2536226053	124.5538882133	140.6564694630
C39	118.2033073439	124.9628117540	139.6433863780
C40	118.5077063784	125.1384843070	138.2877115799
C41	116.8663791117	125.1223033611	140.0458995322
C42	117.5109295539	125.4418342589	137.3543266281
C43	115.8655994899	125.4211504249	139.1234842952
C44	116.1830711009	125.5718519481	137.7701949160
H45	117.6441959504#	129.9707652629#	128.7995438216#
H46	117.8513703777#	129.7498311560#	127.1948927685#

H47	118.9905682527	129.0514672065	128.2564196022
H48	115.9852316005	128.4012646585	128.0578172376
H49	117.1411451693	127.6773014934	126.9685112044
H50	116.4982203049	125.3941355501	127.6221201680
H51	118.0642326113	127.5095935609	130.8974872054
H52	116.8803034895	123.7217602851	129.5057369408
H53	116.3262779294#	129.8431699715#	134.7835685456#
H54	116.3895103171#	129.1526953136#	133.3050841659#
H55	117.6222381223	128.7870693077	134.4246772029
H56	115.9332030158	127.6722601167	135.8654715523
H57	114.6325775334	128.1179121775	134.7658079111
H58	122.0185851408#	125.7875135735#	137.4724879120#
H59	121.1177291589#	126.3387998109#	136.2270055299#
H60	122.7654305202	126.7587123888	136.2815554054
H61	123.3454385138	124.5781463051	135.4297403105
H62	122.0123026785	123.8150113749	136.2680008093
H63	122.7737342011	125.4867634739	132.9630996080
H64	119.4325638987	123.9433876646	134.9822163254
H65	120.8223013656	125.4769343895	131.3250580090
H66	122.0339366760#	121.7673959430#	132.7956241568#
H67	121.9057683667	121.5135222239	131.1253035897
H68	123.3089601323#	121.1488706298#	131.9842082687#
H69	122.3440488252	118.9986907625	131.7009628714
H70	121.9700834807	119.4215481727	133.3603664137
H71	116.5129737704	119.2028173307	131.1061350106
H72	118.2092187802	117.7811525451	129.9646325291
H73	120.6048084611	118.0120022975	130.5302931486
H74	119.6390620627	121.0676125763	133.3946124806
H75	117.2479006716	120.8485267568	132.8244130635
H76	119.7774569207#	125.1411506824#	142.5952910228#
H77	118.8836746251#	126.2880935387#	141.8520972746#
H78	120.5233130503	126.1689446864	141.4506125685
H79	120.1607326769	124.2312438931	140.1323832428
H80	118.8784795332	123.6609857431	141.1737399377
H81	116.6057672767	124.9880351263	141.0933538225
H82	114.8368132060	125.5263245627	139.4571698986
H83	115.3953893775	125.7842856031	137.0524069345
H84	119.5381364822	125.0253904870	137.9583905849
H85	117.7729194011	125.5834285824	136.3080680990
H86	110.2531355067#	122.7250813035#	139.2154086296#
H87	108.7330266949	122.3257593223	138.5699744061
H88	108.9442666266#	123.5248262961#	139.7756956569#
H89	108.6065695423	124.5300000019	137.4861752219
H90	109.9545416717	125.1356235520	138.4307875290
H91	112.2975493451	124.2212396485	138.0047959104
H92	113.9012454753	123.6593267580	136.2181893357
H93	109.0252863104	123.4954472564	135.3184038159
H94	110.6237320831	122.9401039485	133.5164460111
H95	113.0732930218	123.0088909227	133.9469058797
H96	115.0231224083	124.9531584986	133.1901083877
H97	113.9299544873	126.0634994890	134.0049991213
O98	116.1454276540	123.5965970683	132.5051951492
O99	116.7563336381	123.0979013090	133.7105547432
H100	116.2820575986	123.5782595142	134.4224946564

The B3LYP optimized structure in **Figure 3.**

Energies: E= --2137.780813, solv = -0.057642, disp = -82.74 Z_0 = 546.57

Cu1	117.8947714276	124.5102653979	132.3711699050
C2	116.5619978616#	129.0119920872#	134.2799996162#
C3	115.7265834055	127.8219056415	134.7741708093
C4	115.9618944013	126.5681504084	133.9447298882
O5	117.0454555735	126.3853594652	133.3537806032
N6	114.9719094127	125.6511465376	133.8943221944
C7	117.9430030438#	129.3260028128#	128.0959959786#
C8	117.0719022210	128.0381038305	128.0848480250
C9	117.5468104875	126.9115953986	128.9511818894
N10	118.2815721670	125.8481564824	128.4444044560
C11	117.4130454739	126.6323177268	130.2870114886

C12	118.5528006958	124.9802984859	129.4455676204
N13	118.0403462301	125.4346978467	130.5791441245
C14	122.0159981978#	125.9889975822#	136.4929945239#
C15	122.3094657347	124.7118678956	135.6545766737
C16	121.5181298854	124.7414739025	134.3860101566
N17	121.7997762216	125.5804575975	133.3163381511
C18	120.2931499970	124.2037107407	134.0916440569
C19	120.7667555484	125.5370738931	132.4425818972
N20	119.8360356160	124.7106119479	132.8907192646
C21	109.3710041487#	123.1240013782#	138.9649946135#
C22	109.5766273189	124.2455318580	137.9138184936
C23	110.5254427230	123.8921152382	136.7854104433
C24	111.9133647468	123.8963437368	137.0000032665
C25	110.0585310371	123.5624425026	135.5051662776
C26	112.8052451352	123.5819719447	135.9725729129
C27	110.9443473674	123.2500818445	134.4706626918
C28	112.3224655818	123.2594739549	134.6992785975
C29	122.3130017692#	121.1350035774#	132.0729971766#
C30	121.7286149402	119.7251216462	132.2973662437
C31	120.2301655933	119.6971998797	132.0598035428
C32	119.6972465037	119.1032518905	130.9060613368
C33	119.3419895686	120.3091538530	132.9572552736
C34	118.3232449683	119.1172210765	130.6541001862
C35	117.9659139748	120.3253421521	132.7135642077
C36	117.4511114784	119.7290140818	131.5579377732
C37	119.6179977203#	125.6200040618#	141.7319945440#
C38	119.2582650423	124.5460600011	140.6687692912
C39	118.2856598684	124.9582207976	139.5822477863
C40	118.5998462343	124.8175367466	138.2242954845
C41	116.9957393030	125.4013236373	139.9201385862
C42	117.6492727583	125.0745594251	137.2318764566
C43	116.0412355635	125.6566749075	138.9376663673
C44	116.3610196567	125.4797717995	137.5882408283
H45	117.6441959770#	129.9707653930#	128.7995437137#
H46	117.8513702666#	129.7498310630#	127.1948927360#
H47	119.0005153272	129.0868780669	128.2538215641
H48	116.0450244566	128.2912151320	128.3709442334
H49	117.0030502270	127.6623234755	127.0562127670
H50	118.5429368638	125.7247914826	127.4749952100
H51	116.9169832073	127.1949384356	131.0609305248
H52	119.0946712611	124.0551953386	129.3162736337
H53	116.3262780240#	129.8431700235#	134.7835685041#
H54	116.3895103056#	129.1526952480#	133.3050841584#
H55	117.6276760076	128.8044457479	134.4126437213
H56	115.9868359444	127.5817913959	135.8125742976
H57	114.6566776498	128.0651227429	134.7709665271
H58	122.0185851005#	125.7875135448#	137.4724879061#
H59	121.1177291552#	126.3387998331#	136.2270055716#
H60	122.7599422435	126.7693310571	136.2957969363
H61	123.3822516520	124.5970053823	135.4598859763
H62	122.0022715644	123.8192233612	136.2093470821
H63	122.6349971194	126.1404171000	133.2089507373
H64	119.6917561831	123.5139520985	134.6624385270
H65	120.7222003867	126.1047882501	131.5263231958
H66	122.0339367147#	121.7673960272#	132.7956240980#
H67	121.9149758227	121.5322363021	131.1278644363
H68	123.3089601296#	121.1488706035#	131.9842082334#
H69	122.2144027050	119.0130072905	131.6207134149
H70	121.9569050267	119.3888108742	133.3174817969
H71	116.3792711152	119.7153400954	131.3753009284
H72	117.9339937342	118.6378626751	129.7598990053
H73	120.3664885629	118.6157940028	130.2005850827
H74	119.7268602964	120.7656718614	133.8666805385
H75	117.3045602508	120.7949234717	133.4354619974
H76	119.7774568941#	125.1411506792#	142.5952910259#
H77	118.8836746231#	126.2880935357#	141.8520972783#
H78	120.5226850470	126.1683553185	141.4470606090
H79	120.1752562540	124.1582603970	140.2098487480
H80	118.8092153978	123.6897290427	141.1913520660
H81	116.7267378427	125.5152425916	140.9676651566
H82	115.0429509415	125.9765467756	139.2240658533

H83	115.6031300014	125.6384855454	136.8272545750
H84	119.5932055177	124.4742429406	137.9434504232
H85	117.9150655700	124.9460104140	136.1852475513
H86	110.2531354888#	122.7250812900#	139.2154086707#
H87	108.7324800597	122.3267060643	138.5689132427
H88	108.9442665796#	123.5248262592#	139.7756956503#
H89	108.6062730706	124.5431431998	137.4995506530
H90	109.9708454336	125.1305486110	138.4318419995
H91	112.2995452614	124.1490347028	137.9855741077
H92	113.8740482022	123.5712669794	136.1742110282
H93	108.9877981002	123.5549052983	135.3146931148
H94	110.5566125005	122.9959567533	133.4878332333
H95	113.0106312611	122.9951032117	133.8993637991
H96	115.0845743808	124.8379628081	133.2833595770
H97	114.0768534262	125.7968982071	134.3426881750
O98	116.1296052294	123.6590336250	132.1609247818
O99	117.4555342348	123.3636540794	133.8768212120
H100	116.7895106902	123.8224983170	134.4204894145
H101	116.2450201973	122.6922166603	132.1072492536

The B3LYP optimized Cu(III) structure corresponding to the one in **Figure 3.**
Energies: E= --2137.789263, solv = -0.060982, disp = -81.77 Z_0 = 546.95

Cu1	117.8190157088	124.2871148193	132.2772725453
C2	116.5619979765#	129.0119920450#	134.2799996609#
C3	115.7462329887	127.8006752591	134.7608899344
C4	115.9760645477	126.5892010367	133.8661770806
O5	117.0756426948	126.3945211968	133.3162964425
N6	114.9482083251	125.7280720525	133.6873191042
C7	117.9430030536#	129.3260028197#	128.0959960225#
C8	117.1124706757	128.0073371435	128.1337107688
C9	117.6162056276	126.8866767457	129.0079226348
N10	118.3959877658	125.8535501606	128.4985564531
C11	117.4281212594	126.5523556111	130.3274566793
C12	118.6396676385	124.9506471207	129.4734481520
N13	118.0707560776	125.3554861193	130.6005421204
C14	122.0159981323#	125.9889975763#	136.4929944808#
C15	122.2236278186	124.7004101959	135.6324188244
C16	121.3583488221	124.7383497385	134.4038654091
N17	121.5542671950	125.6380196326	133.3616115276
C18	120.1733220320	124.1152235023	134.1036318354
C19	120.5210649180	125.5487579570	132.4968737657
N20	119.6682017583	124.6276533046	132.9224974476
C21	109.3710041742#	123.1240013773#	138.9649945949#
C22	109.5738346532	124.2478604531	137.9160122216
C23	110.5251132781	123.9036721947	136.7870371312
C24	111.9130544300	123.9193845429	137.0016404878
C25	110.0605906559	123.5777249197	135.5050883923
C26	112.8077612277	123.6233856376	135.9716886829
C27	110.9491173752	123.2818377514	134.4680959629
C28	112.3269760087	123.3057410159	134.6966440421
C29	122.3130017572#	121.1350035573#	132.0729971949#
C30	121.7617057814	119.7115072783	132.3047870333
C31	120.2738101605	119.6160136471	132.0282849441
C32	119.8019600687	119.0052681495	130.8569664446
C33	119.3333300209	120.1701439810	132.9093943112
C34	118.4355771541	118.9474286119	130.5720092395
C35	117.9653053428	120.1142070618	132.6314018894
C36	117.5107939274	119.5028988431	131.4594799090
C37	119.6179977535#	125.6200040832#	141.7319945445#
C38	119.2598747791	124.5481521661	140.6666340233
C39	118.2984551592	124.9530023869	139.5651546282
C40	118.5798432274	124.6831746614	138.2183557784
C41	117.0430433998	125.4993231507	139.8797519684
C42	117.6279647075	124.9071137406	137.2189025500
C43	116.0889308040	125.7297354562	138.8896388559
C44	116.3727874130	125.4187554269	137.5565205435
H45	117.6441959911#	129.9707654164#	128.7995436982#
H46	117.8513702494#	129.7498310457#	127.1948927296#

H47	119.0083215998	129.1159272505	128.2506921988
H48	116.0845990850	128.2357269987	128.4356227158
H49	117.0317512715	127.6087776765	127.1144185253
H50	118.7015184840	125.7683865327	127.5376672997
H51	116.8889670431	127.0694147066	131.1031929674
H52	119.1986380761	124.0377521281	129.3318319218
H53	116.3262779505#	129.8431700314#	134.7835684566#
H54	116.3895102760#	129.1526952643#	133.3050841660#
H55	117.6302457620	128.8129512446	134.4070228797
H56	116.0406398088	127.5226630576	135.7800609594
H57	114.6755017500	128.0364745950	134.7961672838
H58	122.0185851264#	125.7875135344#	137.4724879039#
H59	121.1177291602#	126.3387998614#	136.2270055919#
H60	122.7767789946	126.7460824182	136.2766439389
H61	123.2775310583	124.5547376779	135.3667408864
H62	121.9294628540	123.8151820823	136.2051922471
H63	122.3361345546	126.2730772530	133.2693204995
H64	119.6264038502	123.3662025349	134.6519639673
H65	120.4138206392	126.1520489526	131.6105160180
H66	122.0339367221#	121.7673960369#	132.7956240924#
H67	121.9101286735	121.5214719702	131.1258061467
H68	123.3089601289#	121.1488705987#	131.9842082254#
H69	122.2922488539	119.0072915590	131.6538170697
H70	121.9755363730	119.3993601770	133.3356372377
H71	116.4461882159	119.4388769614	131.2495974086
H72	118.0939670959	118.4560377446	129.6649585218
H73	120.5132858429	118.5597787886	130.1649776134
H74	119.6709822808	120.6373521542	133.8320094632
H75	117.2613063780	120.5412456780	133.3387966598
H76	119.7774568730#	125.1411506755#	142.5952910278#
H77	118.8836746151#	126.2880935262#	141.8520972824#
H78	120.5227109969	126.1693122632	141.4484876059
H79	120.1787907623	124.1516046337	140.2187122583
H80	118.8013027908	123.6954138756	141.1877010819
H81	116.7961144760	125.7138240417	140.9167015187
H82	115.1166605702	126.1325014833	139.1608617384
H83	115.6129267234	125.5562102082	136.7927933425
H84	119.5459270290	124.2564264472	137.9555277450
H85	117.8562237774	124.6631662048	136.1842095546
H86	110.2531354829#	122.7250812835#	139.2154086813#
H87	108.7325314608	122.3268052637	138.5686189730
H88	108.9442665774#	123.5248262666#	139.7756956455#
H89	108.6025135552	124.5424101928	137.5017635947
H90	109.9636029639	125.1333297955	138.4365628159
H91	112.2971190370	124.1709625353	137.9882312094
H92	113.8768943227	123.6294225372	136.1716647969
H93	108.9899631677	123.5620533231	135.3146111660
H94	110.5637341395	123.0313979085	133.4833661603
H95	113.0168946823	123.0579156707	133.8927566383
H96	115.0726634507	124.9774468220	133.0040154037
H97	114.0354421457	125.8775816766	134.0954267596
O98	116.1301296485	123.8330800364	131.7534887554
O99	117.5593714253	123.2239859370	133.7201583912
H100	116.6534156823	123.4162137561	134.0208098576
H101	116.0662682107	122.8791625215	131.9454172575

The B3LYP optimized TS structure in **Figure 4.**
Energies: E= --2178.265733, solv = -0.055888, disp = -85.74 Z_0 = 571.59

Cu1	117.8166220000	124.4689420000	132.4356230000
C2	116.5619980000	129.0119920000	134.2800000000
C3	115.6999340000	127.8431530000	134.7868530000
C4	115.9017180000	126.5780050000	133.9693860000
O5	116.9837120000	126.3588320000	133.3894730000
N6	114.8820220000	125.7000570000	133.9031970000
C7	117.9430030000	129.3260030000	128.0959960000
C8	117.0778390000	128.0327100000	128.1002650000
C9	117.5411900000	126.9209160000	128.9941200000
N10	118.4277060000	125.9425000000	128.5633320000

C11	117.2557970000	126.5756880000	130.2917570000
C12	118.6388290000	125.0631360000	129.5712780000
N13	117.9417260000	125.4248490000	130.6372440000
C14	122.0159980000	125.9889980000	136.4929940000
C15	122.3122410000	124.7280210000	135.6291750000
C16	121.5017810000	124.7758270000	134.3710660000
N17	121.6869590000	125.7268350000	133.3747770000
C18	120.3250990000	124.1544420000	134.0441020000
C19	120.6437440000	125.6630460000	132.5130820000
N20	119.8002960000	124.7184550000	132.8965820000
C21	109.3710040000	123.1240010000	138.9649950000
C22	109.5774710000	124.2440180000	137.9126390000
C23	110.5373660000	123.8880620000	136.7955400000
C24	111.9218620000	123.8816630000	137.0285230000
C25	110.0845950000	123.5637130000	135.5094060000
C26	112.8248680000	123.5623870000	136.0133000000
C27	110.9817150000	123.2458060000	134.4867650000
C28	112.3560570000	123.2450530000	134.7342840000
C29	122.3130010000	121.1350040000	132.0729970000
C30	121.7575840000	119.7191110000	132.3301280000
C31	120.2434150000	119.7119710000	132.3854360000
C32	119.4850350000	119.6957820000	131.2044700000
C33	119.5625520000	119.7723530000	133.6083970000
C34	118.0905300000	119.7426960000	131.2445980000
C35	118.1681450000	119.8251210000	133.6554520000
C36	117.4269370000	119.8103310000	132.4725880000
C37	119.6179980000	125.6200040000	141.7319940000
C38	119.2502070000	124.5333240000	140.6808660000
C39	118.2668350000	124.9288700000	139.5985020000
C40	118.6045540000	124.8650600000	138.2409610000
C41	116.9510030000	125.2881580000	139.9361740000
C42	117.6561110000	125.1256550000	137.2480300000
C43	115.9981480000	125.5436330000	138.9517480000
C44	116.3460390000	125.4499080000	137.6009380000
H45	117.6441960000	129.9707650000	128.7995440000
H46	117.8513700000	129.7498310000	127.1948930000
H47	119.0025920000	129.0934400000	128.2535100000
H48	116.0480210000	128.2871410000	128.3732220000
H49	117.0203360000	127.6386640000	127.0773260000
H50	118.8266870000	125.8790380000	127.6361560000
H51	116.6186240000	127.0721760000	131.0059610000
H52	119.2818380000	124.1985820000	129.4965990000
H53	116.3262780000	129.8431700000	134.7835690000
H54	116.3895100000	129.1526950000	133.3050840000
H55	117.6240590000	128.7917970000	134.4176210000
H56	115.9533730000	127.6056260000	135.8289340000
H57	114.6366380000	128.1141450000	134.7816580000
H58	122.0185850000	125.7875130000	137.4724880000
H59	121.1177290000	126.3388000000	136.2270060000
H60	122.7556990000	126.7763940000	136.3038710000
H61	123.3842560000	124.6294300000	135.4198900000
H62	122.0210510000	123.8210730000	136.1689760000
H63	122.4628180000	126.3720830000	133.3097550000
H64	119.7843680000	123.3718980000	134.5530270000
H65	120.5297340000	126.3068100000	131.6544650000
H66	122.0339370000	121.7673960000	132.7956240000
H67	121.8986000000	121.5191940000	131.1302270000
H68	123.3089600000	121.1488700000	131.9842080000
H69	122.1041570000	119.0440460000	131.5378190000
H70	122.1646550000	119.3297140000	133.2715190000
H71	116.3405350000	119.8294700000	132.5096310000
H72	117.5219240000	119.7033750000	130.3182570000
H73	119.9943150000	119.6284150000	130.2450590000
H74	120.1318720000	119.7707130000	134.5357860000
H75	117.6613060000	119.8698600000	134.6147400000
H76	119.7774570000	125.1411510000	142.5952910000
H77	118.8836750000	126.2880930000	141.8520970000
H78	120.5224080000	126.1645740000	141.4398770000
H79	120.1655560000	124.1456270000	140.2186610000
H80	118.8102540000	123.6815060000	141.2178640000
H81	116.6645510000	125.3408620000	140.9841770000

H82	114.9812700000	125.8006470000	139.2368460000
H83	115.5980170000	125.6124450000	136.8298520000
H84	119.6169610000	124.5851970000	137.9587930000
H85	117.9408370000	125.0665790000	136.2002240000
H86	110.2531350000	122.7250810000	139.2154090000
H87	108.7330320000	122.3260240000	138.5697630000
H88	108.9442670000	123.5248260000	139.7756960000
H89	108.6087250000	124.5344350000	137.4896980000
H90	109.9637320000	125.1328460000	138.4300470000
H91	112.2962230000	124.1302150000	138.0196460000
H92	113.8915650000	123.5519430000	136.2245160000
H93	109.0163150000	123.5644000000	135.3054630000
H94	110.6045180000	122.9966780000	133.4982680000
H95	113.0559090000	122.9840040000	133.9427400000
H96	114.9998150000	124.8651480000	133.3271230000
H97	113.9990640000	125.8475860000	134.3733940000
H98	115.4092600000	121.7979180000	129.7477500000
C99	114.6616030000	122.2763670000	130.3801140000
H100	114.0715160000	121.5792960000	130.9738390000
H101	114.0685170000	123.0379080000	129.8741780000
H102	115.3632930000	122.9602250000	131.2380070000
O103	115.9576940000	123.5674480000	132.1276600000
O104	117.5600480000	123.2957990000	133.9203830000
H105	117.1872070000	123.8209510000	134.6479490000
H106	116.2305690000	122.9005480000	132.8202880000

The B3LYP optimized structure in **Figure 4**..
Energies: E= --2178.300647, solv = -0.060450, disp = -89.65 Z_0 = 577.19

Cu1	117.2877569510	124.5027141179	132.2867163028
C2	116.5619981835#	129.0119918135#	134.2799995881#
C3	115.6900769614	127.8385573707	134.7655914897
C4	115.7048335092	126.6303409713	133.8390923467
O5	116.7854103034	126.2344769602	133.3269172406
N6	114.5336960305	126.0249230766	133.6126147514
C7	117.9430034435#	129.3260025502#	128.0959964891#
C8	117.0280175979	128.0740320508	128.0488808509
C9	117.3963072123	126.9871964520	128.9969504656
N10	118.5635474432	126.2521511074	128.8484968815
C11	116.7938909656	126.4692110367	130.1121738743
C12	118.6375885428	125.3385149910	129.8387690646
N13	117.5766518300	125.4506936223	130.6205502961
C14	122.0159982779#	125.9889975647#	136.4929943805#
C15	122.2470397732	124.7041599101	135.6430098243
C16	121.4715720485	124.7979481844	134.3641535597
N17	121.7136173974	125.7888448409	133.4253163699
C18	120.3168149263	124.1908986623	133.9279511182
C19	120.7189163498	125.7479188004	132.4991929605
N20	119.8539701804	124.7890381300	132.7687058592
C21	109.3710045163#	123.1240014509#	138.9649943394#
C22	109.5562475336	124.2386935533	137.9086538283
C23	110.2896936286	123.7968714551	136.6606189844
C24	111.6877334746	123.8850606053	136.5791155536
C25	109.5942554402	123.2745498739	135.5606245008
C26	112.3691989560	123.4690669253	135.4332133978
C27	110.2706435701	122.8550925279	134.4133943758
C28	111.6617998503	122.9534719895	134.3438002177
C29	122.3130008148#	121.1350043627#	132.0729965534#
C30	121.8008169850	119.7073194985	132.3640401787
C31	120.2900668630	119.6207620112	132.4595667125
C32	119.5107562801	119.3495887844	131.3242611750
C33	119.6301410302	119.8171895761	133.6825781085
C34	118.1182275469	119.2610885265	131.4093188401
C35	118.2385252958	119.7331653141	133.7749715876
C36	117.4770505080	119.4479981004	132.6381724380
C37	119.6179979799#	125.6200042411#	141.7319943595#
C38	119.2637294566	124.5469269315	140.6660732459
C39	118.2939582983	124.9675256838	139.5816516317

C40	118.6484388458	124.9548521514	138.2272026630
C41	116.9714410501	125.3030389027	139.9182082143
C42	117.7059939527	125.2319793779	137.2319278280
C43	116.0244571931	125.5738684398	138.9336134709
C44	116.3863636080	125.5238374056	137.5841784343
H45	117.6441958794#	129.9707655892#	128.7995434923#
H46	117.8513700033#	129.7498309420#	127.1948927058#
H47	118.9943997668	129.0644088616	128.2574336698
H48	115.9907869311	128.3673537422	128.2400375102
H49	117.0378618525	127.6617699359	127.0306861389
H50	119.2426939489	126.3693213964	128.1081535985
H51	115.8675425394	126.7593946410	130.5819990287
H52	119.4534571019	124.6475334942	129.9766466274
H53	116.3262777924#	129.8431699979#	134.7835684379#
H54	116.3895102754#	129.1526953821#	133.3050841830#
H55	117.6235870252	128.7878636890	134.4164368960
H56	116.0358216636	127.4964019977	135.7477438088
H57	114.6530965209	128.1694603468	134.8968063309
H58	122.0185849604#	125.7875135562#	137.4724879184#
H59	121.1177291388#	126.3387998817#	136.2270056904#
H60	122.7719024425	126.7538158028	136.2829887181
H61	123.3147832993	124.5316227680	135.4585824399
H62	121.8845141025	123.8244945025	136.1852559021
H63	122.4832703433	126.4438310238	133.4436070405
H64	119.7773964474	123.3686940370	134.3770549790
H65	120.6721328086	126.4327814552	131.6638637631
H66	122.0339373291#	121.7673960807#	132.7956242882#
H67	121.8857124734	121.4961649836	131.1274940265
H68	123.3089601827#	121.1488700761#	131.9842087493#
H69	122.1552322620	119.0286927929	131.5787417782
H70	122.2455750228	119.3515897235	133.3022499815
H71	116.3970030301	119.3531165361	132.7138306755
H72	117.5381593411	119.0183657680	130.5225112640
H73	120.0022994737	119.1763527616	130.3690591662
H74	120.2156240203	120.0147978626	134.5782302893
H75	117.7503925708	119.8756186303	134.7348266069
H76	119.7774567379#	125.1411505897#	142.5952910052#
H77	118.8836745358#	126.2880934290#	141.8520973368#
H78	120.5226338036	126.1700950179	141.4497819996
H79	120.1830647416	124.1647365583	140.2073805025
H80	118.8155294533	123.6882919241	141.1846624258
H81	116.6746427417	125.3182589836	140.9644533683
H82	115.0008257748	125.8043148265	139.2160954142
H83	115.6323832737	125.6884372647	136.8199206994
H84	119.6679094492	124.6997499542	137.9478459296
H85	118.0073212785	125.2111111898	136.1863522669
H86	110.2531354392#	122.7250812866#	139.2154088407#
H87	108.7274694191	122.3257070088	138.5775524193
H88	108.9442665106#	123.5248262056#	139.7756956401#
H89	108.5722174932	124.6380615389	137.6331293027
H90	110.1054734472	125.0692844097	138.3721913988
H91	112.2453690203	124.2839877203	137.4248208666
H92	113.4535615432	123.5363414908	135.3885394104
H93	108.5095169032	123.2022248870	135.6024362977
H94	109.7089437007	122.4561980342	133.5726479257
H95	112.2001456769	122.6422698239	133.4549803009
H96	114.4630351890	125.1459203428	133.0816701662
H97	113.6975643904	126.3373700416	134.0878768524
H98	117.3145145665	122.0300487988	132.0567065569
C99	117.6603189261	122.7788609988	131.3561842354
H100	117.1236800258	122.8525953445	130.4129695480
H101	118.7429009386	122.8400180511	131.2884011661
H102	114.4175578971	122.8042307647	131.8347297092
O103	114.4729652884	123.4224411924	132.5775208223
O104	116.8779339012	123.3868755026	133.7037876694
H105	116.9266015023	123.9863219826	134.4693419599
H106	115.2881013653	123.1844015235	133.1007347227

The B3LYP optimized structure in **Figure 5.**

Energies: E= --2178.371686, solv = -0.051853, disp = -88.28 Z_0 = 578.80

Cu1	117.8979337086	124.7300375713	132.4824226743
C2	116.5619983938#	129.0119916337#	134.2799996349#
C3	115.6761930406	127.8594693455	134.7884995715
C4	116.0009207341	126.5250965569	134.1332725318
O5	117.1736859635	126.2499065295	133.8152138794
N6	114.9921581944	125.6532760179	133.9409049117
C7	117.9430034377#	129.3260026548#	128.0959964622#
C8	116.9998923500	128.0901382762	128.0251116249
C9	117.2812858513	126.9328013587	128.9343572460
N10	117.6191154793	125.6890100872	128.4248549558
C11	117.2541821014	126.7472722709	130.2952506576
C12	117.7755912181	124.8209890558	129.4548625401
N13	117.5600759593	125.4335902470	130.6065464173
C14	122.0159976197#	125.9889973079#	136.4929943071#
C15	122.3183427700	124.6586059551	135.7401169946
C16	121.5073644391	124.5201425787	134.4957541582
N17	121.8720797048	125.0555511441	133.2711151091
C18	120.2293927070	124.0577947467	134.3096247616
C19	120.8291416613	124.9111846046	132.4147066095
N20	119.8138886012	124.3149414635	133.0178806986
C21	109.3710051701#	123.1240014644#	138.9649942360#
C22	109.5984646139	124.2217527346	137.8925515282
C23	110.6138954953	123.8333484301	136.8346668908
C24	111.9859368946	124.0169239746	137.0704680147
C25	110.2280474954	123.2650523859	135.6116908489
C26	112.9405295047	123.6426955393	136.1218956677
C27	111.1770381028	122.8937603529	134.6553041657
C28	112.5389270895	123.0809280811	134.9053397461
C29	122.3130000273#	121.1350046093#	132.0729965447#
C30	121.6909033721	119.7479637256	132.3239637508
C31	120.1753487642	119.8381764371	132.3740664406
C32	119.4327752296	120.0030819026	131.1924275778
C33	119.4788830069	119.7977792713	133.5897421702
C34	118.0419577387	120.1095340731	131.2204917462
C35	118.0852753485	119.8972536587	133.6258541043
C36	117.3582068701	120.0517221720	132.4412210782
C37	119.6179984019#	125.6200043553#	141.7319941886#
C38	119.2591752294	124.5454351530	140.6605955140
C39	118.2039144134	124.9573631917	139.6567438108
C40	118.5362047853	125.4271661215	138.3792200941
C41	116.8429187761	124.8631022910	139.9942814787
C42	117.5434384896	125.7877271508	137.4597526236
C43	115.8458491790	125.2122894703	139.0846563460
C44	116.1918975950	125.6698553248	137.8094140877
H45	117.6441958763#	129.9707655974#	128.7995434836#
H46	117.8513700340#	129.7498309438#	127.1948927037#
H47	118.9851178581	129.0364200795	128.2603316829
H48	115.9687408662	128.4307635598	128.1839534029
H49	117.0187146630	127.7064532978	126.9969110362
H50	117.7097403097	125.4646219632	127.4432616702
H51	117.0375361635	127.4636546960	131.0732231122
H52	118.0384786353	123.7812291675	129.3288398016
H53	116.3262776595#	129.8431699674#	134.7835684260#
H54	116.3895101932#	129.1526954064#	133.3050842011#
H55	117.6184772553	128.7758577352	134.4281275680
H56	115.8165124160	127.7341834093	135.8698608731
H57	114.6151758929	128.0977507281	134.6419903831
H58	122.0185853419#	125.7875136067#	137.4724879182#
H59	121.1177292221#	126.3388001227#	136.2270057263#
H60	122.7660653417	126.7575277602	136.2793149662
H61	123.3896465471	124.5636716368	135.5267206025
H62	122.0666525757	123.8132935070	136.3897551621
H63	122.7660445079	125.4724764241	133.0503863548
H64	119.5714609396	123.5766374866	135.0194108446
H65	120.8476700129	125.2444720850	131.3877786138
H66	122.0339378158#	121.7673962182#	132.7956243562#
H67	121.9097429157	121.5371868400	131.1326006308
H68	123.3089602064#	121.1488697867#	131.9842089713#

H69	121.9942959679	119.0536478921	131.5304924825
H70	122.0665819064	119.3303183816	133.2658272012
H71	116.2702894304	120.0666629289	132.4620690611
H72	117.4874833464	120.2021222578	130.2900455988
H73	119.9516180612	120.0236027928	130.2363572706
H74	120.0315239633	119.6655441510	134.5170174314
H75	117.5646437411	119.8204548856	134.5768476113
H76	119.7774564461#	125.1411505726#	142.5952910496#
H77	118.8836744749#	126.2880933546#	141.8520973808#
H78	120.5238342789	126.1678974684	141.4510302147
H79	120.1679095797	124.2359833872	140.1316024131
H80	118.8952543997	123.6501218120	141.1797814624
H81	116.5645029314	124.4979934696	140.9802695859
H82	114.8004080379	125.1259189141	139.3675283574
H83	115.4113757979	125.9340480289	137.1020251933
H84	119.5827747288	125.5147671539	138.0994570405
H85	117.8262280129	126.1648960690	136.4790693206
H86	110.2531352332#	122.7250808983#	139.2154089482#
H87	108.7334105219	122.3229177101	138.5748355389
H88	108.9442664464#	123.5248264996#	139.7756954612#
H89	108.6458488368	124.4836459402	137.4174073727
H90	109.9505405537	125.1315034598	138.3965254627
H91	112.3069952027	124.4580613087	138.0125890433
H92	113.9986955307	123.7788501203	136.3330557356
H93	109.1710122390	123.1143359001	135.4044392084
H94	110.8509052520	122.4559729278	133.7156779594
H95	113.2822328326	122.7807330597	134.1704206894
H96	115.2058271512	124.7234391826	133.5785186879
H97	114.0463179970	125.8487958320	134.2379031611
H98	117.5290179624	122.0077859213	137.7971011138
C99	116.8717677578	121.9892633803	136.9205864264
H100	115.8356465139	122.1453071447	137.2421674552
H101	116.6434410986	122.3039188286	132.9233275088
O102	116.3595794332	123.1198520468	133.3723154460
O103	117.2797731924	122.9696633843	135.9597974670
H104	117.2595130325	123.8427477269	136.3939307884
H105	116.6744144993	123.0165237002	134.3065545833
H106	116.9509024891	121.0135901113	136.4368475002

The B3LYP optimized TS structure in **Figure 7.**
Energies: E= --2194.325504, solv = -0.059285, disp = -86.34 Z_0 = 566.07

Cu1	117.8039660000	124.4679200000	132.4980520000
C2	116.5619980000	129.0119920000	134.2800000000
C3	115.6913280000	127.8430920000	134.7773370000
C4	115.9031680000	126.5940920000	133.9386030000
O5	117.0384060000	126.3254260000	133.4962980000
N6	114.8421810000	125.8059490000	133.6863690000
C7	117.9430030000	129.3260030000	128.0959960000
C8	117.0862710000	128.0268690000	128.1136880000
C9	117.5519610000	126.9297260000	129.0260240000
N10	118.4756820000	125.9750250000	128.6211650000
C11	117.2387530000	126.5743230000	130.3150260000
C12	118.6783400000	125.1001120000	129.6353010000
N13	117.9429490000	125.4414810000	130.6815420000
C14	122.0159980000	125.9889980000	136.4929940000
C15	122.3285630000	124.7264090000	135.6401180000
C16	121.5246000000	124.7546480000	134.3793550000
N17	121.7367020000	125.6576040000	133.3460360000
C18	120.3259210000	124.1618510000	134.0822270000
C19	120.6859090000	125.5935210000	132.4921800000
N20	119.8130230000	124.6941040000	132.9153070000
C21	109.3710040000	123.1240010000	138.9649950000
C22	109.5794760000	124.2436240000	137.9130030000
C23	110.5234270000	123.8815190000	136.7838510000
C24	111.9116520000	123.8777340000	136.9947640000
C25	110.0510690000	123.5498610000	135.5067720000
C26	112.7993730000	123.5556980000	135.9669920000
C27	110.9327780000	123.2280980000	134.4718560000
C28	112.3110470000	123.2314660000	134.6970340000

C29	122.3130010000	121.1350040000	132.0729970000
C30	121.7995070000	119.7032840000	132.3303710000
C31	120.2895200000	119.5694660000	132.2761900000
C32	119.6728150000	118.7322730000	131.3344130000
C33	119.4694010000	120.2731470000	133.1714800000
C34	118.2846560000	118.5769510000	131.3047620000
C35	118.0815140000	120.1295660000	133.1489520000
C36	117.4840690000	119.2685910000	132.2193690000
C37	119.6179980000	125.6200040000	141.7319940000
C38	119.2535940000	124.5426840000	140.6680390000
C39	118.2263780000	124.9384480000	139.6270280000
C40	118.5572650000	125.0615630000	138.2723160000
C41	116.8846130000	125.1268340000	139.9981720000
C42	117.5818160000	125.3432580000	137.3109970000
C43	115.9047130000	125.4023300000	139.0466540000
C44	116.2484320000	125.5009850000	137.6952160000
H45	117.6441960000	129.9707650000	128.7995440000
H46	117.8513700000	129.7498310000	127.1948930000
H47	119.0040180000	129.0992550000	128.2536560000
H48	116.0532460000	128.2775300000	128.3776640000
H49	117.0368970000	127.6176690000	127.0961450000
H50	118.8987710000	125.9193960000	127.7043420000
H51	116.5620940000	127.0513060000	131.0055450000
H52	119.3434190000	124.2510010000	129.5768810000
H53	116.3262780000	129.8431700000	134.7835680000
H54	116.3895100000	129.1526950000	133.3050840000
H55	117.6218550000	128.7848860000	134.4200910000
H56	115.9475100000	127.5959110000	135.8150340000
H57	114.6304170000	128.1212890000	134.7723400000
H58	122.0185850000	125.7875130000	137.4724880000
H59	121.1177290000	126.3388000000	136.2270060000
H60	122.7522110000	126.7800190000	136.3059530000
H61	123.4027500000	124.6378330000	135.4381170000
H62	122.0437920000	123.8216100000	136.1868800000
H63	122.5342340000	126.2715210000	133.2503820000
H64	119.7552140000	123.4278070000	134.6287600000
H65	120.5912470000	126.2045970000	131.6078620000
H66	122.0339370000	121.7673960000	132.7956240000
H67	121.8968450000	121.5077740000	131.1264270000
H68	123.3089600000	121.1488700000	131.9842090000
H69	122.2448040000	119.0211180000	131.5969610000
H70	122.1577830000	119.3681830000	133.3140260000
H71	116.4081200000	119.1032490000	132.2388150000
H72	117.8332120000	117.9000500000	130.5839160000
H73	120.2889150000	118.1798420000	130.6286480000
H74	119.9190710000	120.9325500000	133.9090150000
H75	117.4819850000	120.6941410000	133.8560200000
H76	119.7774570000	125.1411510000	142.5952910000
H77	118.8836750000	126.2880930000	141.8520970000
H78	120.5230550000	126.1671740000	141.4465340000
H79	120.1654580000	124.1973840000	140.1670930000
H80	118.8584170000	123.6650970000	141.1968920000
H81	116.6030860000	125.0328700000	141.0447600000
H82	114.8705460000	125.5275750000	139.3566800000
H83	115.4780320000	125.6872350000	136.9518360000
H84	119.5900050000	124.9177770000	137.9635300000
H85	117.8683930000	125.4405930000	136.2655380000
H86	110.2531350000	122.7250810000	139.2154090000
H87	108.7321080000	122.3266150000	138.5695200000
H88	108.9442670000	123.5248260000	139.7756960000
H89	108.6094660000	124.5456980000	137.5010280000
H90	109.9802340000	125.1266820000	138.4292610000
H91	112.3009270000	124.1322950000	137.9787310000
H92	113.8697400000	123.5502660000	136.1607460000
H93	108.9797130000	123.5488180000	135.3191780000
H94	110.5402900000	122.9742900000	133.4904020000
H95	112.9990880000	122.9714060000	133.8953390000
H96	114.9729150000	124.9755530000	133.1019740000
H97	113.9281580000	125.9929260000	134.0744560000
H98	116.5364540000	121.0660460000	130.8264330000
N99	115.9223270000	121.8125150000	130.4742120000

H100	114.9696580000	121.4466440000	130.3979580000
H101	115.9969190000	122.7985320000	131.0873930000
O102	115.9669900000	123.5861740000	132.1064660000
O103	117.5623810000	123.2818990000	133.9968340000
H104	117.3097540000	123.8154540000	134.7691910000
H105	116.1966070000	123.0591290000	132.9217190000

The B3LYP optimized structure directly after the TS.

Energies: E= --2194.338154, solv = -0.058785, disp = -86.47 Z_0 = 568.70

Cu1	117.7560328417	124.4755515753	132.4374144751
C2	116.5619980294#	129.0119918135#	134.2799995509#
C3	115.6911954559	127.8410908208	134.7733211275
C4	115.8568512783	126.6079894160	133.9005052000
O5	116.9792440357	126.3101478217	133.4413088167
N6	114.7649134276	125.8676173587	133.6356864886
C7	117.9430032884#	129.3260027419#	128.0959961316#
C8	117.0730167061	128.0366432885	128.0970965192
C9	117.5327581601	126.9303613666	128.9965495466
N10	118.4575966414	125.9796110247	128.5846912316
C11	117.2229586599	126.5717018920	130.2838826043
C12	118.6646899417	125.1017625026	129.5944626857
N13	117.9311155958	125.4397849486	130.6428966098
C14	122.0159984474#	125.9889977655#	136.4929944566#
C15	122.2812119946	124.7027441284	135.6547281451
C16	121.4602870552	124.7266302469	134.4033418337
N17	121.6928866856	125.6090130191	133.3569854942
C18	120.2561857285	124.1427006010	134.1029532315
C19	120.6505738513	125.5438913974	132.4939294726
N20	119.7628336006	124.6618545272	132.9208416777
C21	109.3710043038#	123.1240014385#	138.9649945287#
C22	109.5755259049	124.2462252857	137.9149663191
C23	110.5286683692	123.9030034900	136.7866999095
C24	111.9160927138	123.9047448209	137.0071748267
C25	110.0678625196	123.5986901843	135.4983141885
C26	112.8135414227	123.6199150128	135.9766406548
C27	110.9597082251	123.3126158171	134.4608696372
C28	112.3369471521	123.3250611411	134.6943685595
C29	122.3130009967#	121.1350042852#	132.0729965802#
C30	121.7957270162	119.7030493560	132.3114586808
C31	120.2929593512	119.5550727494	132.1645284627
C32	119.7494645615	118.5895464364	131.3047333970
C33	119.4070777337	120.3664931146	132.8912232265
C34	118.3673176911	118.4183589604	131.1921320131
C35	118.0248239547	120.2044814902	132.7858925804
C36	117.4991784114	119.2203435538	131.9395752007
C37	119.6179981451#	125.6200042676#	141.7319943759#
C38	119.2567745694	124.5485541521	140.6637219539
C39	118.2584133229	124.9599816818	139.6000951094
C40	118.5935275352	124.9818779382	138.2404911680
C41	116.9312731234	125.2526961327	139.9583580162
C42	117.6320228237	125.2568209816	137.2625912235
C43	115.9650910982	125.5202239007	138.9905988812
C44	116.3097646779	125.5099815117	137.6355889850
H45	117.6441958655#	129.9707654368#	128.7995436261#
H46	117.8513701532#	129.7498310456#	127.1948927395#
H47	119.0014937200	129.0898969788	128.2541571840
H48	116.0422055887	128.2931338831	128.3639678924
H49	117.0203285867	127.6397808899	127.0749362398
H50	118.8821147855	125.9313873992	127.6680000349
H51	116.5475344213	127.0441556309	130.9783166044
H52	119.3308730296	124.2539587936	129.5322671790
H53	116.3262778316#	129.8431699705#	134.7835685014#
H54	116.3895103211#	129.1526953737#	133.3050841738#
H55	117.6219815787	128.7846603070	134.4196932049
H56	115.9746438365	127.5673168413	135.7964163376
H57	114.6346269315	128.1333015342	134.8056531892
H58	122.0185849370#	125.7875134550#	137.4724878881#

H59	121.1177290968#	126.3387997393#	136.2270056452#
H60	122.7660574617	126.7611979411	136.2888494589
H61	123.3486856279	124.5802658223	135.4351969371
H62	121.9828079630	123.8158861548	136.2232069127
H63	122.4989707762	126.2116447981	133.2604422015
H64	119.6794129042	123.4149585938	134.6514075751
H65	120.5723709371	126.1419043574	131.5992824484
H66	122.0339372216#	121.7673959757#	132.7956243389#
H67	121.9019801634	121.5148481294	131.1269324340
H68	123.3089601815#	121.1488701748#	131.9842087508#
H69	122.2889204464	119.0186993318	131.6113479365
H70	122.0980312638	119.3742943685	133.3162639362
H71	116.4249033974	119.0549347218	131.8916422399
H72	117.9720591608	117.6463353849	130.5369897836
H73	120.4168401964	117.9528215612	130.7285624266
H74	119.7941111922	121.1281555493	133.5620850678
H75	117.3780977954	120.8482935971	133.3736745753
H76	119.7774566629#	125.1411506093#	142.5952910299#
H77	118.8836745070#	126.2880934007#	141.8520973202#
H78	120.5227750350	126.1688981337	141.4483436423
H79	120.1718388231	124.1832120795	140.1832370134
H80	118.8326168786	123.6796158705	141.1850745406
H81	116.6471940741	125.2400304669	141.0082566255
H82	114.9403046587	125.7218524672	139.2906954777
H83	115.5465424993	125.6840607732	136.8827809214
H84	119.6155948365	124.7590767984	137.9431668883
H85	117.9188070651	125.2722610930	136.2131959400
H86	110.2531354655#	122.7250812680#	139.2154087187#
H87	108.7326693019	122.3264334718	138.5690331181
H88	108.9442665461#	123.5248262381#	139.7756956430#
H89	108.6049316856	124.5412981823	137.4992965856
H90	109.9645333053	125.1321417494	138.4355879380
H91	112.2971677681	124.1385634001	137.9994760550
H92	113.8821915570	123.6184855696	136.1780869470
H93	108.9979591337	123.5937474815	135.3027600458
H94	110.5763530595	123.0798292018	133.4708457684
H95	113.0310815862	123.0897630962	133.8906038495
H96	114.8530228754	125.0493562356	133.0307398975
H97	113.8646122761	126.0731984642	134.0459287683
H98	116.6668245796	120.8819658360	130.2963067403
N99	115.9412841000	121.5925617465	130.1199692562
H100	115.2722837388	121.1554128556	129.4721403915
H101	115.7763175971	123.0053607612	131.2598848358
O102	115.7866901638	123.6483359720	132.0207661723
O103	117.4350842780	123.2427478967	133.8688550568
H104	117.2187948034	123.7330040293	134.6789267101
H105	116.0713803094	123.1358528426	132.8348630821

The B3LYP optimized structure **in Figure 9.**
Energies: E= --2194.362105, solv = -0.054773, disp = -88.01 Z_0 = 571.74

Cu1	118.2449583377	124.9778755631	132.5680611445
C2	116.5619980985#	129.0119916795#	134.2799995742#
C3	115.7087077523	127.8180809251	134.7463683010
C4	116.1415113277	126.5339921323	134.0585884355
O5	117.3585747230	126.2915468648	133.9182732129
N6	115.1851888436	125.6933023017	133.6256505386
C7	117.9430033723#	129.3260026787#	128.0959963620#
C8	117.0083947053	128.0857726759	128.0360178601
C9	117.3572728807	126.9583126312	128.9504358640
N10	117.8909081303	125.7693929945	128.4829299621
C11	117.2899650527	126.7938874067	130.3106907634
C12	118.1189989479	124.9500185469	129.5398527259
N13	117.7636992247	125.5456762794	130.6652461963
C14	122.0159978135#	125.9889974172#	136.4929943583#
C15	122.3967614063	124.6699608867	135.7667428447
C16	121.6999276339	124.5182058166	134.4617547946
N17	122.1356154893	125.0728425555	133.2689589352

C18	120.4453659355	124.0390321645	134.1932804597
C19	121.1491044428	124.9292452664	132.3486628407
N20	120.1056490676	124.3121714358	132.8823811006
C21	109.3710050955#	123.1240014417#	138.9649942524#
C22	109.6130412289	124.2176817353	137.8901109811
C23	110.6953906460	123.8503320285	136.8929887108
C24	111.9735902629	124.4188881876	136.9812729706
C25	110.4579869546	122.9215943725	135.8661785956
C26	112.9804968856	124.0788867647	136.0729180441
C27	111.4601239485	122.5739861587	134.9588872312
C28	112.7291128765	123.1542782931	135.0555788692
C29	122.3130001404#	121.1350046359#	132.0729964317#
C30	121.7378271882	119.7264872178	132.3234464830
C31	120.2216226772	119.7395727853	132.2914331504
C32	119.5207644421	119.3037884752	131.1568043122
C33	119.4815903474	120.2278949352	133.3784080105
C34	118.1262329863	119.3619350241	131.1006136075
C35	118.0869804642	120.2897682782	133.3283188728
C36	117.4019934534	119.8590357962	132.1869394198
C37	119.6179983889#	125.6200043950#	141.7319942527#
C38	119.2386404395	124.5467704399	140.6650212163
C39	118.1133833607	124.9501153100	139.7372032088
C40	118.3376601323	125.2441956417	138.3860717205
C41	116.7980574280	125.0400276285	140.2243151958
C42	117.2880529677	125.6331036509	137.5431032239
C43	115.7463333370	125.4230565297	139.3936508111
C44	115.9878564412	125.7216220055	138.0486498223
H45	117.6441958685#	129.9707655501#	128.7995435236#
H46	117.8513700858#	129.7498309777#	127.1948927144#
H47	118.9873252938	129.0419623456	128.2586724003
H48	115.9784609472	128.4066084267	128.2320028702
H49	117.0021384478	127.6995785565	127.0085521308
H50	118.0551846683	125.5429197692	127.5113147966
H51	116.9342694564	127.4885628210	131.0563361300
H52	118.5284089200	123.9545209314	129.4504305299
H53	116.3262777956#	129.8431699616#	134.7835684993#
H54	116.3895102913#	129.1526953874#	133.3050841810#
H55	117.6224748184	128.7889924292	134.4248558573
H56	115.8168573734	127.6570968512	135.8256495538
H57	114.6436289573	128.0060251113	134.5624840408
H58	122.0185852616#	125.7875135698#	137.4724879108#
H59	121.1177291819#	126.3388000057#	136.2270057081#
H60	122.7528819374	126.7756709727	136.2947102465
H61	123.4850571454	124.6017424081	135.6517191827
H62	122.1066372041	123.8180975499	136.3916680819
H63	123.0398214266	125.4949971772	133.1077847836
H64	119.7459790617	123.5534289643	134.8594405022
H65	121.2264116839	125.2754845649	131.3288086869
H66	122.0339377207#	121.7673961358#	132.7956243916#
H67	121.9030932642	121.5226157483	131.1301956275
H68	123.3089602107#	121.1488698148#	131.9842090232#
H69	122.1156149132	119.0317348845	131.5647021940
H70	122.0877609929	119.3523429394	133.2943381969
H71	116.3148328663	119.8738874503	132.1582812134
H72	117.6055947802	119.0068904061	130.2156026743
H73	120.0745915894	118.9057834065	130.3095601641
H74	119.9999596017	120.5560825095	134.2765519717
H75	117.5323712697	120.6583960009	134.1860574630
H76	119.7774564508#	125.1411505426#	142.5952910321#
H77	118.8836744918#	126.2880933725#	141.8520973844#
H78	120.5240874622	126.1619562189	141.4429535319
H79	120.1220311858	124.2735284919	140.0771899109
H80	118.9332371535	123.6317407895	141.1890039688
H81	116.5991678236	124.8087380301	141.2682772735
H82	114.7393862905	125.4945453645	139.7951058573
H83	115.1710460902	126.0548103880	137.4151009382
H84	119.3500242535	125.1937993464	137.9909145141
H85	117.4997815723	125.8733496777	136.5051507138
H86	110.2531352583#	122.7250809374#	139.2154089221#
H87	108.7313589418	122.3241253126	138.5768412591
H88	108.9442664744#	123.5248264964#	139.7756954775#

H89	108.6774683192	124.4385056664	137.3618815364
H90	109.9070815845	125.1462506711	138.3945448120
H91	112.1777565280	125.1452142166	137.7651232496
H92	113.9569564249	124.5548200564	136.1504180503
H93	109.4708900019	122.4747908077	135.7707814256
H94	111.2485481045	121.8553703762	134.1719541555
H95	113.5116559301	122.8888589891	134.3480233363
H96	115.4561748599	124.7945325746	133.2177818603
H97	114.2058018716	125.8833942920	133.7865376384
H98	116.3935275860	121.5862432886	136.2727092662
N99	116.4982891320	122.4824532920	135.7914306566
H100	115.8048057181	123.1149399975	136.2045628167
H101	116.7905255043	122.4928300242	132.4413766228
O102	116.5189169161	123.2255456373	133.0195570439
O103	117.8070984099	122.9661895639	136.1162023896
H104	117.7760230775	123.3500346555	137.0142295723
H105	116.5815524436	122.8584420611	133.9387319864
